# Interaction between immuno-stem dual lineages in jaw bone formation and injury repair

**DOI:** 10.3389/fcell.2024.1359295

**Published:** 2024-03-06

**Authors:** Ziyi Liu, Xutao Luo, Ruoshi Xu

**Affiliations:** State Key Laboratory of Oral Diseases and National Center for Stomatology and National Clinical Research Center for Oral Diseases and Department of Cariology and Endodontics, West China Hospital of Stomatology, Sichuan University, Chengdu, China

**Keywords:** jawbone, stem cells, immune cells, bone regeneration, signaling pathways, cytokines

## Abstract

The jawbone, a unique structure in the human body, undergoes faster remodeling than other bones due to the presence of stem cells and its distinct immune microenvironment. Long-term exposure of jawbones to an oral environment rich in microbes results in a complex immune balance, as shown by the higher proportion of activated macrophage in the jaw. Stem cells derived from the jawbone have a higher propensity to differentiate into osteoblasts than those derived from other bones. The unique immune microenvironment of the jaw also promotes osteogenic differentiation of jaw stem cells. Here, we summarize the various types of stem cells and immune cells involved in jawbone reconstruction. We describe the mechanism relationship between immune cells and stem cells, including through the production of inflammatory bodies, secretion of cytokines, activation of signaling pathways, etc. In addition, we also comb out cellular interaction of immune cells and stem cells within the jaw under jaw development, homeostasis maintenance and pathological conditions. This review aims to eclucidate the uniqueness of jawbone in the context of stem cell within immune microenvironment, hopefully advancing clinical regeneration of the jawbone.

## 1 Introduction

The jaw is located in the human craniofacial jaw and is divided into maxilla and mandible. The mandible is the only movable bone on the human cranial face, which is inseparable from the temporomandibular joint and the special structure of the mandibular condyle (BI et al., 2023). Alveolar bone is the bone surrounding the teeth in the jaw. The Alveolar bone is also an important periodontal tissue component. Jawbone and periodontal tissues are interrelated. Periodontal tissue inflammation leads to resorption of alveolar bone, which further leads to loss of jaw bone. The health of periodontal tissue is conducive to maintaining jaw homeostasis and the microenvironment of the jaw can promote the regeneration of periodontal tissue (GUIGLIA et al., 2013; ZHU et al., 2023).

The jawbone is a unique bone tissue. Compared to other bones in the body, the jawbone has a faster remodeling rate (JEYARAMAN et al., 2023), which is closely related to the stem cells of the jawbone and its unique immune microenvironment (LUO et al., 2022). Jaw stem cells possess a remarkable proliferative capacity and osteogenic differentiation potential (PAJARINEN et al., 2019). These stem cells play crucial roles in jawbone development, maintenance and regeneration (LOPES et al., 2018). Clinically, jaw inflammation, trauma, and tumors can lead to jaw defects. Ideal jawbone defect restoration is clinically expected during bone inflammation and remodeling. Based on their osteogenic differentiation and proliferative abilities of jaw stem cells, they hold promise as essential tools for treating jaw defects, offering innovative ideas and directions for future treatments.

The jawbone is consistently exposed to a microbe-rich oral environment, resulting in the immune microenvironment of the jaw is different from other bone tissues (LIN and YUAN, 2022). For example, a higher proportion of macrophages are expressed in the jaw (LIN et al., 2021). Immune cells play a critical role in shaping the immune microenvironment of the jawbone and promoting reconstruction or resorption (TAKAYANAGI, 2007). Cytokines secreted by the immune cells can lead to bone reconstruction or loss (YANG and LIU, 2021). The reconstruction and loss of the jawbone are intricately linked to jaw stem cell function (LOPES et al., 2018). Hence, we propose that the involvement of immune cells in bone remodeling or absorption is linked to their capacity to regulate jaw stem cells.

Interactions between immune cells and jaw stem cells occur via diverse mechanisms (TAKAYANAGI, 2007). Several studies that immune cells can affect jaw stem cells either directly or indirectly via cytokine secretion, activation of signaling pathways, and other mechanisms, promoting jaw injury repair. Recent studies have revealed a novel mechanism by which immune cells regulate jaw stem cell function via exosomes (SILVA et al., 2017; LI Z. et al., 2021). MSCs can also exert their immunomodulatory functions through these mechanisms.

Jaw stem cells and immune cells interact during embryonic development and accompany the body throughout its life (TSUKASAKI and TAKAYANAGI, 2019). During development, abnormalities on either side can result in jaw deformity (OKAMOTO et al., 2017). After jaw development completion, jaw stem cells and immune cells jointly maintain jaw homeostasis and adapt to normal physiological and abnormal pathological processes of the body (MUIRE et al., 2020; SALHOTRA et al., 2020; XIONG et al., 2022). We describe the related diseases to illustrate the crucial role of the immune system in jaw development, homeostasis maintenance, and repair in pathological states. In short, the interaction between immune cells and jaw stem cells can help constract a normal jaw.

## 2 Introduction of stem cells in the jaw

**FIGURE 1:**
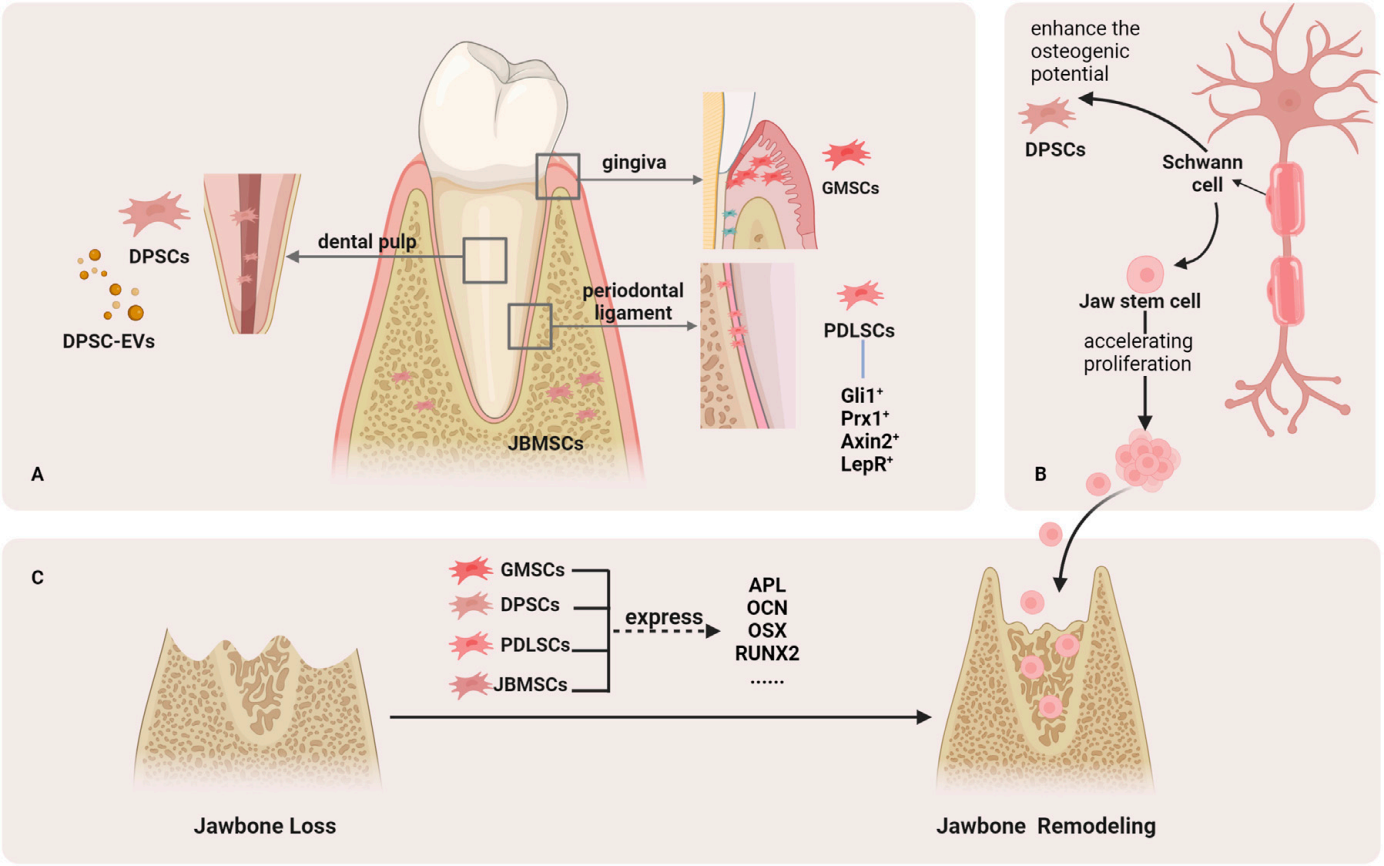
Stem cells that help regenerate the jawbone. **(A)**. Stem cells in the jaw include DPSCs from the dental pulp, GMSCs from the gingiva, PDLSCs from the periodontal tissue, and JBMSCs from the jawbone. **(B)**. Schwann cells can help the reconstruction of jawbone. **(C)**. Stem cells in jaw can help repair jaw defects by expressing osteogenic markers.

There are many kinds of stem cells in the jaw. Studies have reported that the types of stem cells in the oral and maxillofacial regions include periodontal ligament stem cells (PDLSCs) originating from the periodontal ligament, dental pulp mesenchymal stem cells (DPSCs) obtained from dental pulp tissue, and jaw mesenchymal stem cells (JBMSCs) in the jawbone (TATULLO et al., 2015; TOMOKIYO et al., 2019; LI P. et al., 2023). Compared with stem cells derived from long bones, jaw stem cells exhibit a higher rate of proliferation and greater osteogenic potential (JEYARAMAN et al., 2023). Based on the advantages of stem cells in jaw, stem cell therapy has emerged as an effective approach to maintaining jaw bone development, reconstruction, and regeneration (TATULLO et al., 2015; TOMOKIYO et al., 2019; LI P. et al., 2023). Currently, there are many genetic mouse models that can carry out the research of stem cells *in vivo* through lineage tracing (Table 1). Moreover, the regeneration of jawbone is also dependent on the regulation of nerve tissue (CARR et al., 2019). Among them, Schwann cells play an key role in jaw repair (KAUKUA et al., 2014; LOPEZ-LEAL and COURT, 2016). During inflammation, the jaw balance is disrupted, and the inflammatory tissue recruits important cells. These cells actively participate in maintaining jaw balance (Figure 1).

**TABLE 1:** Preclinical/lab models for bone tissue engineering and molecular mechanistic studies.

Name	Application	References
Gli1-CreERT2; Rosa26tdTomato mice	A mouse model of lineage tracing Gli1^+^ cells	SHALEHIN et al. (2022)
Axin2-CreERT2; R26RtdTomato mice	A mouse model of Axin2^+^ cell were labeled	XIE et al. (2022)
Axin2-CreERT2; R26RDTA; R26RtdTomato mice	A mouse model of Axin2^+^ cell were ablated	XIE et al. (2022)
Prx1-cre; R26RtdTomato mice	A mouse model of Prx1^+^ cell were labeled	GONG et al. (2022)
Lepr-CreER; tdTomato mice	A mouse model of Lepr^+^ cell were labeled	ZHANG et al. (2023a)
PLP-CreERT2; Rosa26tdTomato mice	A mouse models of Schwann cell were labeled	JONES et al. (2019)
LysM-Cre mice	A mouse model of Myeloid cell lineages (monocytes, mature macrophages, granulocytes) were labeled	SOLIS et al. (2019)
FoxP3-DTR mice	A mouse model of Treg cell depletion	WANG et al. (2021)
CD11c-DTR mice	A mouse model of DC cells deficiency	ZHAO et al. (2023)
CD19-Cre mice	A mouse model of B cells deficiency	ZENG et al. (2021)
ZDC-DTR mice	A mouse model of DC cell were ablated	ELSAYED et al. (2020)

### 2.1 Periodontal ligament stem cells (PDLSCs)

PDLSCs are key components in the periodontal tissue engineering of seed cells. Compared to other stem cells in the jaw, PDLSCs appear to be the most effective stem cell-based therapy for repairing the alveolar bone and PDL (LI Q. et al., 2020). In particular, PDLSCs activity is regulated by mechanical stimulation (MEN et al., 2020).

The osteoblastic potential of PDLSCs has always been a hot topic. Various types of periodontal ligament stem cells have been identified, including Gli1^+^ cells, Prx1^+^ cells, Axin2^+^, and LepR^+^ cells. Gli1^+^ cells contribute to alveolar bone regeneration, and the activity of Gli1^+^ PDLSCs is regulated by physiological occlusal force (MEN et al., 2020). Gli1 is also a transcription factor required for Hedgehog (Hh) signaling, which helps regulate bone formation (SHALEHIN et al., 2022). Axin2^+^ cells help maintain periodontal tissue homeostasis and facilitate bone healing (LI Q. et al., 2020). Bone morphogenetic protein (BMP) signaling regulates the role of Axin2^+^ cells in periodontal tissue development. The conditional knockout of BMP1A damages Axin2^+^ cells, leading to periodontal defects and alveolar bone loss (XIE et al., 2022). Prx1^+^ cells are also important PDLSC isotypes associated with osteogenesis. An experimental study showed that Prx1^+^ cells are indispensable during PDL reconstruction of transplanted teeth (GONG et al., 2022). Finally, LepR^+^ cells are activated after tooth extraction and contribute to newly formed bone in the extraction socket (ZHANG D. et al., 2020). And LepR^+^ cells can contribute to periodontal homeostasis via Piezo1-mediated mechanosensing (ZHANG D. et al., 2023).

Moreover, MSCs derived from gingiva (GMSCs) have characteristics similar to those of PDLSCs in periodontal tissues, both of which exhibit high potential for osteogenic differentiation (SHEN et al., 2023). When GMSCs were implanted into an animal model of mandibular defects, they repaired the mandible and promoted new bone formation (WANG et al., 2011). GMSCs-derived exosomes can reduce the inflammatory response of PDLSCs (SUN et al., 2022).

### 2.2 Dental pulp mesenchymal stem cells (DPSCs)

DPSCs exhibit superior proliferative capabilities and display enhanced senescence resistance (MA et al., 2019). In addition, opportunely differentiated DPSCs showed an osteoblastic phenotype with the expression of the osteogenic marker genes ALP, RUNX2, and OCN during this process (LEE et al., 2019).

Following pulp trauma, DPSCs increase osteogenic potential through NF-kB pathway or Wnt/β-catenin signaling pathway activation (WANG et al., 2013; ZHANG et al., 2019). Extracellular vesicles (EVs) are secreted by cells. When DPSC-EVs were injected into a mandible defect model, bone formation was observed, which was comparable to the effect of BMP-2 on inducing bone formation (IMANISHI et al., 2021; LEE et al., 2023). Therefore, DPSC-EVs can provide a safe and effective cell-free treatment for jawbone regeneration.

### 2.3 Jawbone mesenchymal stem cells (JBMSCs)

JBMSCs originating from the jawbone demonstrate superior proliferative ability compared to marrow mesenchymal stem cells (BMSCs) (HUANG et al., 2020). Two types of cells were implanted into the periodontal tissue defect models. BMSCs only show osteogenic potential, whereas JBMSCs show cementoid, periodontal ligament-like tissue, and alveolar bone regeneration (NAKAJIMA et al., 2014). Consequently, JBMSCs are particularly well-suited for repairing jaw defects when compared to BMSCs. Implantation of autologous JBMSCs into bone defects can effectively treat jaw defects caused by maxillary cysts (REDONDO et al., 2018). JBMSCs also promote implant osseointegration by inducing osteogenic differentiation (YAN et al., 2023).

A recent study identified a distinct osteogenic progenitor derived from JBMSCs that highly expressed protocadhern Fat4 (Fat4 cells) (JIN et al., 2023). They have significant osteogenic abilities and promote osteogenic differentiation of JBMSCs. Above, we mentioned the DPSC-EVs. Under the action of DPSC-EVs, the osteogenic differentiation potential of JBMSCs is enhanced, thereby aiding jaw regeneration (LEE et al., 2023).

### 2.4 Schwann cells

The interaction between the nerve and bone tissue has been demonstrated during bone repair (CARR et al., 2019). Schwann cells derived from neural crest cells or mesenchymal cells are glial cells of the peripheral nervous system that have important potential for repair and regeneration (LOPEZ-LEAL and COURT, 2016).

Schwann cells play an crucial role in regulating the bone microenvironment and that the repair of jaw defects is dependent on the repair potential of Schwann cells (SALHOTRA et al., 2020; WANG et al., 2023). The lack of Schwann cells directly leads to impaired mandibular healing (JONES et al., 2019). Schwann cells accumulate in damaged alveolar bone and promote bone healing, which is related to jaw stem cell proliferation acceleration (ITOYAMA et al., 2020; ZHANG X. et al., 2023). In addition, Schwann cell-derived extracellular vesicles can enhance the osteogenic potential of DPSCs (WANG et al., 2022). Therefore, the application of Schwann cells in jaw defects is expected to provide a new method for promoting bone regeneration by regulating the nervous system.

### 2.5 Other stem cell in jaw

Stem cells derived from human exfoliated deciduous teeth (SHED), suture-derived mesenchymal stem cells (SuSC), and synovial mesenchymal stem cells (SMSC) from temporomandibular joint also exist in jaw. SHED has the potential of osteogenic differentiation and has superior proliferative (KUNIMATSU et al., 2018). SuSC can quickly migrate to the injured site and promote bone repair when the craniofacial bone is injured (MARUYAMA, 2019). SMSC has the potential of multidirectional differentiation and can differentiate into osteoblasts, chondrocytes, neurons, which is conducive to the repair of mandibular tissue (KOYAMA et al., 2011).

Undoubtedly, there are many more stem cells in the jaw than those mentioned above. Stem cells from different sources in the jaw play their own roles in maintaining the balance of jaw.

## 3 Unique immune microenvironment of the jaw

**FIGURE 2:**
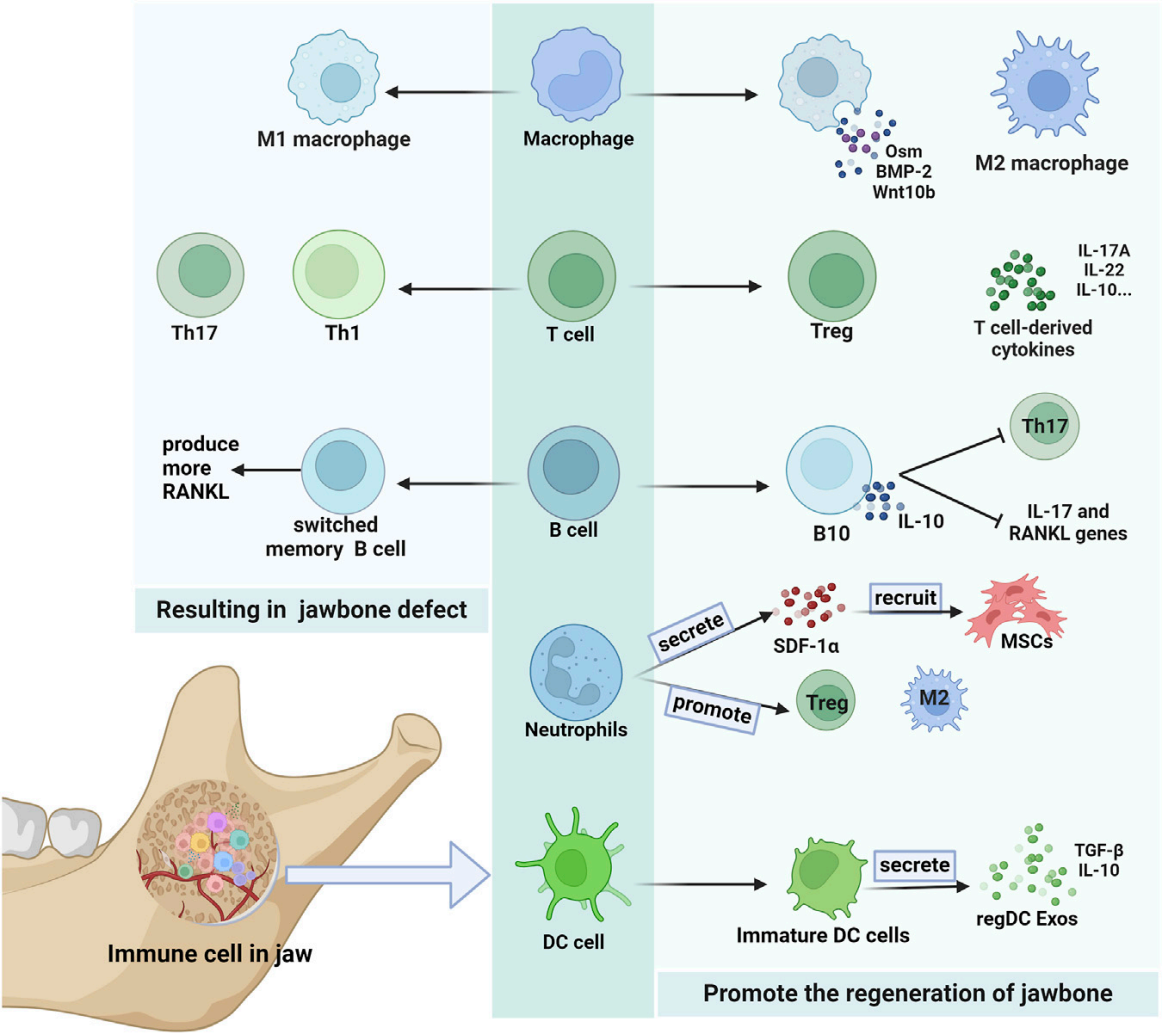
Immune cells in the jaw. There are many kinds of immune cells in the jaw, which can have different effects on the jaw. Some secretions produced by macrophages and T cells promote the jawbone regeneration. B cells that secrete IL-10 also help regenerate the jawbone. Neutrophils in jaw play an important role in the recruitment of MSCs, and can also increase the expression of M2 macrophages and Treg cells to promote bone remodeing. RegDC Exos produced by immature DC cells promote osteogenesis. And Th17 cells and T1 cells are more likely to result jawbone defect. Switched memory B cells can also cause jawbone loss by producing more RANKL.

The jaw is prolonged exposured to a microbiota-rich oral environment (LIN and YUAN, 2022). Therefore, the immune microenvironment of jaw bone is also unique. Compared to long bones, the immune microenvironment of the jawbone is more active, featuring a greater proportion of mature immune cells (LIN et al., 2021). The immune cells in the jaw perform various functions to maintain bone homeostasis. Here, we describe the major immune cells present in the jaw (Figure 2).

### 3.1 Macrophages: key immune cells that regulate jaw regeneration

Macrophages can recruit and regulate MSC differentiation, which plays a key role in bone regeneration (PAJARINEN et al., 2019). Compared to long bones, the jaw exhibits a higher proportion of activated macrophage subpopulations, with these macrophages displaying an enhanced capacity to induce MSC proliferation and migration (LIN et al., 2021). The macrophage in the jaw is an important reason for its unique immune microenvironment.

Macrophages are the most important group of immune cells that interact with MSCs (PAJARINEN et al., 2019). The different effects of macrophages on the jawbone depend on the cytokines they secrete and their polarization. Macrophages express BMP-2, oncostatin M (OSM) and Wnt10b, which mediate osteogenic differentiation of MSCs through paracrine signaling (ZHANG et al., 2022).

There are two main types of macrophages. M1-type macrophages cause bone tissue damage by releasing TNF-α, IL-12, and IL-23, while M2-type macrophages promote sustained bone regeneration by secrete IL-10 and TGF-β (SHAPOURI-MOGHADDAM et al., 2018). Stem cells in the jaw can influence the polarization of macrophages. DPSCs and GMSCs can induce the polarization of M2-type macrophages (ZHANG et al., 2010; YANG et al., 2023). PDLSCs can downregulate the TNF-α expression and upregulate IL-10 and CD163 to promote the polarization of M2-type macrophages (LIU et al., 2019).

### 3.2 T cell: different T cell subsets perform different functions

T cell-mediated adaptive immune responses can recruit MSCs and thus promote tissue regeneration (ZHAO et al., 2020). Regulatory T cells (Treg cells) and T cell-derived cytokines have also been demonstrated to play a positive role in bone regeneration (LI et al., 2018; YU et al., 2022).

The jawbone exhibits a higher proportion of Treg cells than the long bones (KWACK et al., 2021). Many studies have shown that PDLSCs, GMSCs, and DPSCs promote the polarization of Tregs and inhibit the polarization of Th17 cells (YANG et al., 2018; ZHENG et al., 2019; MATSUMURA-KAWASHIMA et al., 2021). And enhancement of the Treg/Th17 ratio can effectively improve bone loss (YANG et al., 2021). In a study on palatal expansion (LI J. et al., 2020), Treg cells were observed to maintain high levels in the later stages, downregulating osteoclast numbers by inhibiting Th1 and Th17 cells and participating in forming a new maxilla. In addition, T Cell-Derived IL-22 and IL-17A can promote bone regeneration by accelerating BMSC osteogenesis (ONO et al., 2016; YU et al., 2022).

### 3.3 B cells: the effect on jaw is double-sided

B cells participate in the bone repair process and play important regulatory roles in the later bone repair stages (KöNNECKE et al., 2014). Studies have shown that jawbone marrow-derived cells have a higher B cell proportion than long bone (KWACK et al., 2021).

In periodontal inflammation states, B cells produce higher TGF-β1 levels, inhibiting osteoblast activity (CHEN et al., 2023). Switched memory B cells produce more RANKL (HAN et al., 2018), leading eventually to alveolar bone loss. Conversely, B cells help regenerate the bone. Zeng et al. discovered that a lack of B cells exacerbats alveolar bone loss (ZENG et al., 2021). Moreover, IL-10-secreting B cells reduce the expression of IL-17 and RANKL genes and inhibit Th17 cell proliferation, thereby reducing alveolar bone uptake (SHI et al., 2020). Consequently, the role of B cells in promoting or inhibiting bone loss remains unclear and warrants further research to elucidate their divergent effects on jaw stem cells.

### 3.4 Neutrophils: play the important function of recruiting MSCs

Many neutrophils are present in the jaw. GMSCs increase the proportion of neutrophils in the immune environment (GAO et al., 2023). Neutrophils are widely believed to have anti-inflammatory, antibacterial, and tissue-repair properties (LIEW and KUBES, 2019; LIN et al., 2021). However, limited research has been conducted on bone tissue repair.

Neutrophils indirectly influence bone regeneration by recruiting important immune cells (ABARICIA et al., 2021). A recent study has shed light on the direct role of neutrophils in bone regeneration, which is required during the initial stages of bone regeneration (CAI et al., 2021). At specific IL-8 thresholds, neutrophils are recruited and undergo to acquire the anti-inflammatory N2 phenotype. N2- programmed neutrophils secrete SDF-1α to induce chemotaxis of BMSCs (WANG et al., 2021). The recruited BMSCs then trigger a regenerative cascade. In addition, BMSCs effectively inhibit neutrophil apoptosis by producing IL-6, IFN-β and granulocyte macrophage colony-stimulating factor (GM-CSF) (CASSATELLA et al., 2011). Neutrophils can also promote the expression of M2-type macrophages and Tregs, thereby indirectly contributing to regeneration (WANG et al., 2021).

### 3.5 Dendritic cells: maintain and restore jaw integrity

The jawbone’s prolonged exposure to an oral microbiota-rich environment renders dendritic cells (DCs) more susceptible to microbial invasion (EL-AWADY et al., 2022). These cells are essential in maintaining the integrity of both the oral mucosa and the mandible (HOVAV, 2014).

DCs are indispensable for immune responses in the jawbone. PDLSCs cause DCs to skew towards a more immature phenotype, which is more conducive to he osteogenic differentiation of MSCs and jaw regeneration (YANG et al., 2019; ASHOUR et al., 2020). Moreover, Immune-regulatory exosome derived from tolerant dendritic cells (regDC Exo), which is abundant in TGF-β/IL-10, exhibits the ability to suppress alveolar bone loss and jaw inflammation (ELASHIRY et al., 2020). DCs also promoted BMSCs recruitment (ZHAO et al., 2023). Ranya et al. discovered that DCs deficiency leads to bone necrosis in the jaw after tooth extraction (ELSAYED et al., 2020).

## 4 Molecular interactions between immune cells and stem cells within the jaw

Multiple mechanisms exist for the interaction between jaw stem cells and immune cells, regulating either jaw regeneration or absorption and helping to maintain jaw homeostasis. Jaw stem cells can regulate immunity, which is conducive to maintaining the jaw immune environment stability. In this section, we focus on the mechanism of the interaction between jaw stem cells and immune cells. These mechanisms include paracrine and signaling pathways.

### 4.1 Interactions between cells occur through the secretions

#### 4.1.1 Role of TNF-α: not only cause bone resorption

Primarily secreted by T cells and macrophages, TNF-α is a critical cytokine in bone immunology, with roles in bone metabolism and remodeling. TNF-α is generally considered that stimulates osteoclast proliferation and leads to bone resorption (YAO et al., 2021). However, the ability of TNF-α to lead bone resorption is variable.

TNF-α exerts unique effects to promote osteogenesis. Preconditioning of MSCs with TNF-α enhanced the immunomodulatory properties of MSCs while increasing the osteogenic differentiation of MSCs (LIN et al., 2017). TNF-α exhibits unique stimulation to GMSCs in jaw. Studies indicate that early exposure to TNF-α enhances GMSC proliferation, and this positive impact wanes with prolonged inflammation (ZHANG F. et al., 2017). And TNF-α stimulates the secretion of exosomes from GMSCs and induces M2 macrophage polarization (WATANABE et al., 2022). GMSC-Exos significantly reduce the number of osteoclasts while promoting preosteogenic cell migration and osteogenic differentiation to help repair jaw defects (JIANG and XU, 2020; WATANABE et al., 2022). In addition, TNF-α can help recruit MSCs. T cells, stimulated by TNF-α, release a large amount of C-Cmotif chemokine ligand 5 to recruit MSCs (ZHAO et al., 2020).

#### 4.1.2 Dual action of interleukins on bone

Interleukins constitute a diverse group of cytokines secreted by the immune cells, each playing a specific role. Interleukins derived from immune cells not only lead to bone loss, but also have a positive effect on bone reconstruction.

Interleukins can affect the osteogenic differentiation of MSCs to mitigate bone loss and aid in bone regeneration. B10 cells, as IL-10 competent B cells, reduce osteoclast generation and inhibit jaw loss by up-regulating IL-10 (CAO et al., 2021). IL-10 can induce the polarization of M2 macrophages, enhance the expression of osteogenic genes BMP2 and ALP in MSCs, and trigger osteogenesis of MSCs thereby promoting bone regeneration (MAHON et al., 2020; LI et al., 2022). IL-35 inhibits bone resorption induced by TNF-α (PENG et al., 2018). IL-35 also can reduce jaw loss by modulating the Th17/Treg balance and reducing the RANKL/OPG ratio (CAFFERATA et al., 2020; LI S. et al., 2023). In addition, a certain IL-6 dose can increase APL activity and the mRNA expression of osteogenic markers and promote osteogenic differentiation of PDLSCs (PURWANINGRUM et al., 2023). IL-6 activates the STAT3 signaling pathway, a critical signaling pathway for bone reconstruction, to promote the osteogenic differentiation of MSCs (XIE et al., 2018). Several studies have shown that IL-17 inhibits osteoblastogenesis. Liao et al. reported that IL-17 and bone cells play synergistic roles in the osteogenic differentiation of MSCs (LIAO et al., 2020). Interleukins also impact the immunomodulatory ability of MSCs. Preconditioning of MSCs with IL-1, significantly enhances their immune-regulatory function, and induces a reduction in the secretion of inflammatory mediators (REDONDO-CASTRO et al., 2017; WONG et al., 2023).

In conclusion, interleukins play a key role in the immune environment of the jawbone and provide an important direction for treating bone diseases.

#### 4.1.3 Significant osteogenic properties of IFN-γ

Interferons are important cytokines produced by immune cells. The cytokine IFN-γ is widely recognized for its osteogenic properties, whereas INF-α and INF-β are acknowledged as anti-osteogenic cytokines (AMARASEKARA et al., 2021). Here we focus on IFN-γ, which is beneficial to MSC osteogenic differentiation and bone formation.

Various immune cells, including B cells, M1 macrophages, and DCs, produce IFN-γ to inhibit osteoclast production (TANG et al., 2018). IFN-γ plays a positive role in maintaining bone homeostasis. IFN-γ–producing T cells can induce osteoblast differentiation by inducing the expression of Runx2, Osterix, Alp, and Bglap (MARUHASHI et al., 2015). In addition, many studies have shown that IFN-γ has a unique effect on DPSCs, dependent on its concentration (SONODA et al., 2016; HE et al., 2017). Moreover, DPSCs treated by IFN-γ release factors that contribute to the homing of BMSCs (STROJNY et al., 2015). However, IFN-γ does not absolutely promote osteogenesis, and it can lead to bone resorption (TAN et al., 2021). It may be due to differences in the concentration of IFN-γ, and the exposure time and the osteoclast differentiation stage (TANG et al., 2018). Clinically, the full use of IFN-γ in different periods and different stages of the function can help to treat jaw defects.

#### 4.1.4 Inflammasomes: affect bone loss or remodeling

**FIGURE 3:**
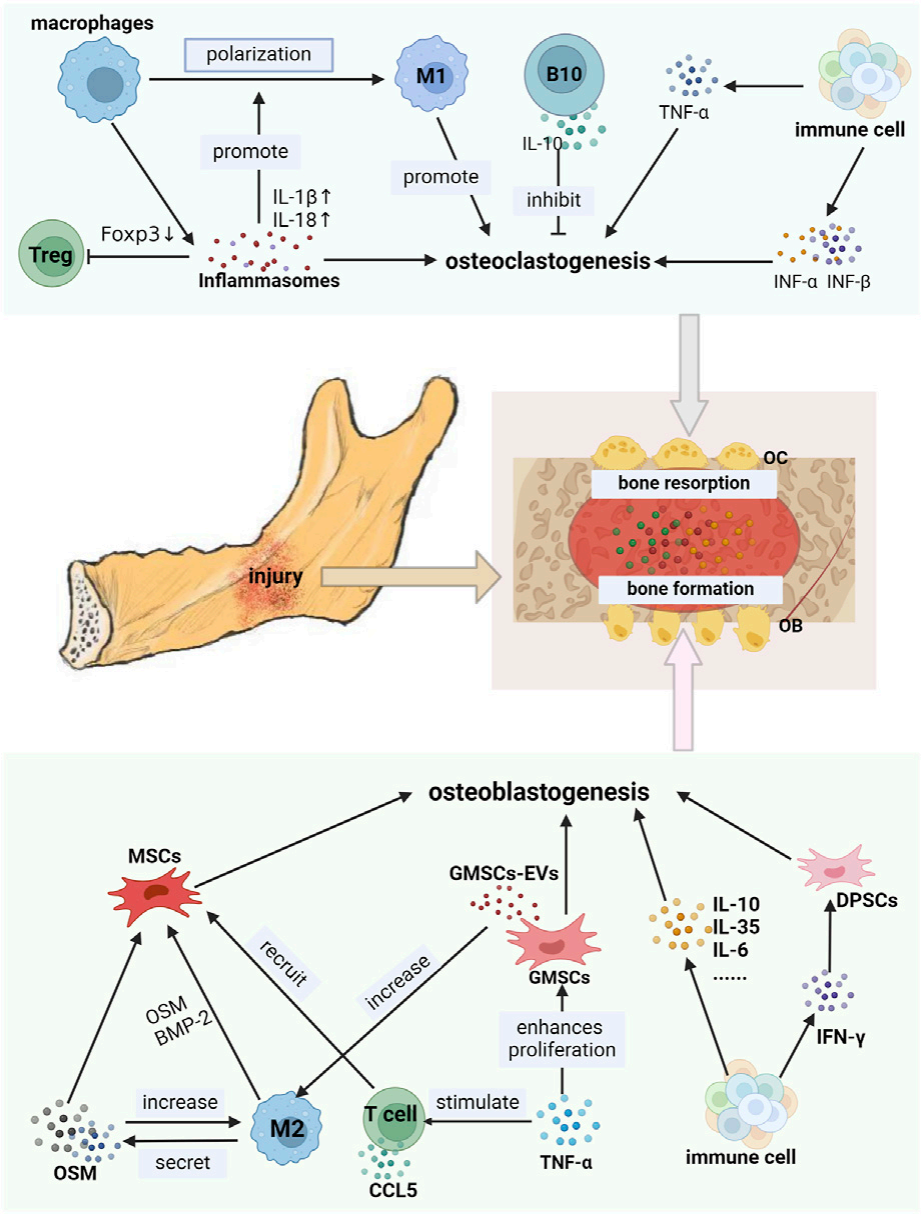
Immune cells interacted with jaw stem cells by secreta. OB: osteoblast, OC osteoclasts, CCL5: C-Cmotif chemokine ligand 5. B10: B cells secreting IL-10. The secretions produced by immune cells in the jawbone exhibit a dual effect on the jawbone. As shown in the picture, M2-type macrophages secrete OSM and BMP-2. TNF-α stimulates the production of GMSCs-Evs by GMSCs. Interleukins such as IL-10, IL-6, and IL-35 are secreted by various immune cells along with IFN-γ. All of these can promote the osteoblastogenesis of jaw stem cells, thereby facilitating the bone formation. CCL5 secreted by T cells can recruit MSCs. On the contrary, inflammasomes generated by macrophages, IFN-α and IFN-β can enhance osteoclastogenesis of jaw bone stem cells and exacerbate defects in the jaw bone.

Inflammasomes are supramolecular protein complexes that facilitate the secretion and maturation of pro-inflammatory cytokines and the activation of inflammatory responses. Inflammasome activation regulates the activities of various cells within the jawbone, thereby affecting bone loss and reconstruction (Figure 3).

In an inflammatory state, the activation of NLRP3 inflammasomes in macrophages upregulate cytokines IL-1β and IL-18 levels, promoting M1-type macrophage polarization (LI Y. et al., 2021). NLRP3 inflammasomes can disrupt the Treg/Th17 ratio by directly inhibiting Foxp3 expression, resulting in increased expression of the inflammatory factors such as IL-17, IL-10, and IL-4, thereby promoting jawbone loss (LENG et al., 2023). The NLRP3 inflammasomes recruit polymorphonuclear neutrophils (PMN) to periodontal lesions. Subsequently, neutrophils can participate in the induction of inflammatory jawbone loss (HAJISHENGALLIS et al., 2016; CHEAT et al., 2022). These studies indicate that inflammasomes promote bone loss, but appropriate inflammasome stimulation is conducive to bone regeneration. The osteoblast differentiation process stimulated by BMP7 requires appropriate inflammasome activity (SARTORETTO et al., 2019). In the inflammatory state, inflammasome suppression can lead to the rapid spread of bacteria, resulting in increased bone damage (VON MOLTKE et al., 2013). Consequently, reasonable use of the properties of inflammasomes is expected for treating jawbone loss.

### 4.2 Generate cellular interactions through signaling pathways

#### 4.2.1 Piezo1: Stimulates jaw regeneration through mechanical signals

Mechanical signals, similar to biological and chemical stimuli, affect cellular functions (SOLIS et al., 2019). The Piezo1 signaling pathway is a mechanosensitive ion channel that plays a vital role in bone formation and mechanoload-dependent remodeling (SUN et al., 2019). Piezo1 is observed in various cell types, including osteoblast lineage cells and innate immune cells (LI et al., 2019; ORSINI et al., 2021).

One study found that activation of Piezo1 signaling can promote the osteogenic differentiation of MSCs by up-regulating the expression of osteogenic factor BMP2 (SUGIMOTO et al., 2017). Piezo1 signaling disruption leads to reduced bone mass and heightened bone resorption (WANG L. et al., 2020). During jaw reconstruction, the proliferation of macrophages is linked to the activation of the Piezo1 signaling pathway (XU et al., 2022). Mechanical stimulation activates the Piezo1 signaling pathway, causing macrophages to polarize toward the M2 phenotype, thus promoting bone formation by BMSCs (CAI et al., 2023). In addition, DCs can sense mechanical stimulation of the bone tissue through Piezo1 and perform appropriate functions to aid in tissue repair (CHAKRABORTY et al., 2021).

#### 4.2.2 TGF-β signaling aids in jaw development and bone regeneration

TGF-β signaling is pivotal for bone maintenance and essential for jaw development. Research indicates that the proliferation and maturation of immature osteoblasts are tightly linked to TGF-β signaling (PETERS et al., 2017).

TGF-β plays a regulatory role in the capacity of immune cells to promote osteogenesis in jaw stem cells. M2 macrophages secrete TGF-β1 to boost osteogenesis in BMSCs (CAI et al., 2023). And active TGF-β produced by immune cells can recruit MSC (MAO et al., 2020). The osteogenic differentiation of BMSCs can be enhanced by IL-4-loaded hydrogel scaffold through TGF-β1/Smad pathway activation (ZHANG J. et al., 2020). In addition, TGF-β-mediated expression of Msx1 induces jaw stem cell proliferation (OKA et al., 2007). Loss of TGF-β signal results in failure to cure and incomplete mandible development. BMP are members of the TGF-β superfamily (THIELEN et al., 2019). BMP-2 accelerates the differentiation of MSCs into osteoblasts (HALLORAN et al., 2020). The M2 macrophages can stimulate bone formation by releasing BMP-2 (ZHOU and GRAVES, 2022).

#### 4.2.3 Wnt plays a critical role in stimulating osteogenic differentiation

The Wnt signaling pathway influences bone health and is critical for the osteogenic differentiation of MSCs. The classical Wnt signaling pathway mediated by β-catenin is the most well-characterized.

Activation of the Wnt signaling pathway promotes the osteogenic differentiation of MSCs. Triggering the Wnt/β-catenin signaling pathway induces osteogenic differentiation of PDLSCs (CAO et al., 2017). SHED-Exos derived from human exfoliated deciduous teeth carry Wnt3a and activate the classical Wnt signaling pathway, thereby enhancing the osteogenic differentiation of PDLSCs (WANG M. et al., 2020). In the context of Axin2+ cells, which were discussed earlier, the action of Wnt3a substantially improves bone healing (YUAN et al., 2018).

Immune cells affect jaw stem cells via the Wnt signaling pathway. Macrophages are an important source of Wnt ligands during inflammation and healing, and T cells produce Wnt10b (WEITZMANN, 2017). IL-6 derived from immune cells mediates the osteogenic differentiation of hPDLSCs via the Wnt signaling pathway (PURWANINGRUM et al., 2023). In the presence of lithium chloride (a Wnt activator), macrophages are recruited to periodontal defect sites. Macrophages stimulate the osteogenic differentiation of PDLSCs and significantly upregulate the mRNA expression of osteogenic markers (Runx2 and OCN) (ZHENG et al., 2022), which promote jaw defects healing.

#### 4.2.4 Hedgehog (Hh) maintains jaw tissue homeostasis

Hh signaling pathway is closely related to embryonic development, tissue homeostasis, and stem cell maintenance. Proteins involved in bone formation include sonic hedgehog (Shh) and Indian hedgehog (Ihh) (ZHOU and GRAVES, 2022).

The Hh pathway promotes the osteogenic differentiation of jaw stem cell. This is essential for the growth of the temporomandibular joint (TMJ) (SHIBUKAWA et al., 2007). The ciliary protein (Ift88) participates in chondrogenesis and bone formation during mandibular development by modulating Shh signaling (KITAMURA et al., 2020). Guan et al. reported that bone formation by BMSCs is inhibited under high glucose conditions; however, the addition of recombinant Shh alleviates this inhibition (GUAN et al., 2009).

**FIGURE 4:**
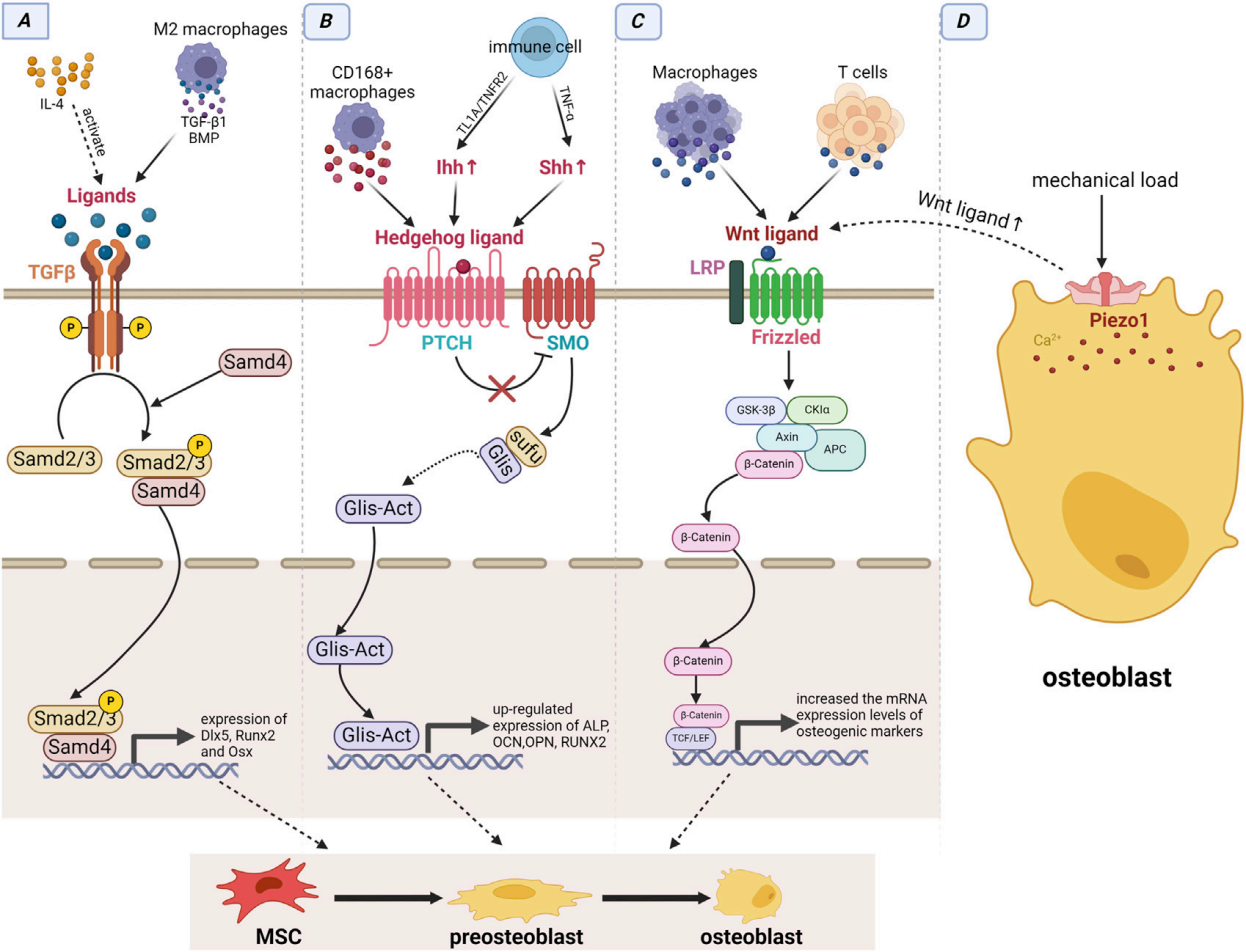
Immune cells interacted with jaw stem cells by signaling pathways. **(A)** TGF-β signaling pathway: The immune cell-derived IL-4 can activate ligands of TGF-β signaling pathway, and macrophages can directly produce ligands to activate this pathway. **(B)** Hedgehog signaling pathway: Immune cells can upregulate Ihh and Shh via the TL1A/TNFR2 axis and secreting TNF-α, while CD168+ macrophages can directly express ligands of the Hh signaling pathway. **(C)** Wnt/β-catenin signaling pathway: Both T cells and macrophages can produce ligands for this pathway. **(D)** Piezo1 signaling pathway: Under mechanical load, the pathway is activated and can promote the expression of ligands of the wnt signaling pathway. Immune cells triggers the activation of these four signaling pathways, which subsequently enhance the expression of osteogenic factors and facilitate the osteogenic differentiation of MSCs through intracellular signal transduction.

Immune cells can affect the action of MSCs through the Hh signaling pathway. D S et al. reported that TNF-α, continuously secreted by macrophages, was crucial for activating Shh signaling (GHORPADE et al., 2013). Shh signaling can stimulate PDLSC proliferation. Gli1s is an important transcription factor in the Hh signaling pathway. Previously, we mentioned that Gli1^+^ cells are a type of PDLSCs that contribute to alveolar bone regeneration (HOSOYA et al., 2020; SHALEHIN et al., 2022). CD168+ macrophages can also directly express ligands activated by the Hh signaling pathway (VALVERDE LDE et al., 2016). The impact of Hh signaling on PDLSCs may be attributed to the activation of Gli1^+^ cells, thus fostering bone proliferation. TL1A is expressed in various immune cells, such as monocytes, macrophages, dendritic cells, and T cells. The TL1A/TNFR2 axis enhances the immunomodulatory effects of stem cells by upregulating the Ihh signaling pathway (AL-AZAB et al., 2021). The classical Hh signaling pathway relies on primary cilia for transduction, which is indispensable for bone development and repair. Moreover, primary ciliary signals are observed in immune cells (TAO et al., 2023) (Figure 4).

#### 4.2.5 Interplay of signaling pathways in jaw defect repair

The signaling pathways in the jaw are not isolated and are interrelated and jointly govern jaw regeneration and reconstruction.

Piezo1 upregulates Wnt1 expression, a key stimulatory signal for activating of Wnt signaling pathway (LI et al., 2019). Recently, Hu et al. reported that Piezo1 activation promotes osteogenic differentiation of Gli1^+^ MSCs by activating the Wnt/β-catenin signaling pathway (HU et al., 2023). Moreover, TGF-β/BMP signal and Hh signal have been confirmed to have a synergistic effect on the osteogenic differentiation of MSCs, activation of both increases osteogenic potential of MSCs (VAN DER HORST et al., 2003; REICHERT et al., 2013). The relationship between the Hh and Wnt signaling pathways is particularly complex. Activation of the Wnt signaling pathway inhibiting Hh signaling, by promoting the expression of Gli3, an Hh signaling antagonist (ALVAREZ-MEDINA et al., 2008). However, Hu et al. reported that Hh-induced osteogenesis requires activated Wnt signaling (HU et al., 2005). In conclusion, the Piezo1, Wnt, TGF-β, and Hh signaling pathways interact with each other to maintain jaw homeostasis.

### 4.3 EVs from immune cells affect jaw stem cells function

Cells produce and release various populations of EVs that contain bioactive molecules. Serving as key messengers in intercellular communication, EVs can deliver bioactive substances to recipient cells and induce their corresponding effects.

EVs from immune cells affect jaw stem cell function. Macrophage-derived EVs increase the expression of osteoblast differentiation markers and promote MSC osteogenesis via the BMP2/Smad5 pathway (LIU et al., 2020). EVs can recruit MSCs. One study showed that DC-derived EVs facilitate MSC recruitment by enriching molecules, such as osteopontin and MMP-9, which are involved in cell recruitment (SILVA et al., 2017). Exosomes are nanovesicular structures in extracellular vesicles released by cells. Studies have demonstrated that Immune cell-derived exosomes regulate the proliferation, differentiation, and migration of jaw stem cells. M0-type, M1-type, and M2-type macrophage-derived exosomes exert different effects on bone repair (KANG et al., 2020). Exosomes derived from M2 macrophages (M2D-Exos) promote osteogenic differentiation and reduce lipogenic differentiation of BMSCs by upregulating miR-690, IRS-1, and TAZ (LI Z. et al., 2021). M2D-Exos contains high IL-10 mRNA levels, which ultimately upregulate IL-10 expression in BMSCs (CHEN et al., 2022). As mentioned previously, IL-10 aids in the osteogenic differentiation of BMSCs. Treatment with DC-derived exosomes increases Runx2 expression and APL activity (WANG et al., 2014). Therefore, DC-derived exosomes can induce the osteogenic differentiation of BMSCs.

EVs are important mediators of these interactions. BMSC-derived small extracellular components secrete exosomes that possess anti-inflammatory and regulatory functions (HA et al., 2020). MSC-EVs can impede DC maturation, and immature DC can promote the osteogenic differentiation of MSCs (REIS et al., 2018; YANG et al., 2019). Liu et al. reported that BMSC-EVs modulate TGF-β1 expression and macrophage polarization to regulate inflammatory immune responses (LIU et al., 2021). These studies have demonstrated that MSC-EVs ultimately lead to bone regeneration by regulating immune cells. Zheng et al. reported that PDLSC-EVs participate in the regulation of the Th17/Treg balance by targeting SIRT1 (ZHENG et al., 2019). Jana et al. reported that GMSC-EVs can reduce the secretion of pro-inflammatory factors by mononuclear macrophages and T cells, and inhibit T cell activation while inducing Treg formation (ZARUBOVA et al., 2022). DPSC-EVs promote M2-type macrophage polarization by inhibiting TLR and NFκΒ signaling (ZHENG et al., 2020). Additionally, exosomes derived from JBMSCs promoted M2-type macrophage polarization by targeting pknox1 with miR-233 (HE et al., 2019).

Overall, EVs can promote jaw tissue regeneration and repair, regulate the immune response, and may lead to jaw resorption. Consequently, EVs have various potential applications in treating jaw defects and are expected to become important therapeutic tools.

## 5 Cellular interaction of immune cells and stem cells in jaw development, homeostasis, and repair

**FIGURE 5:**
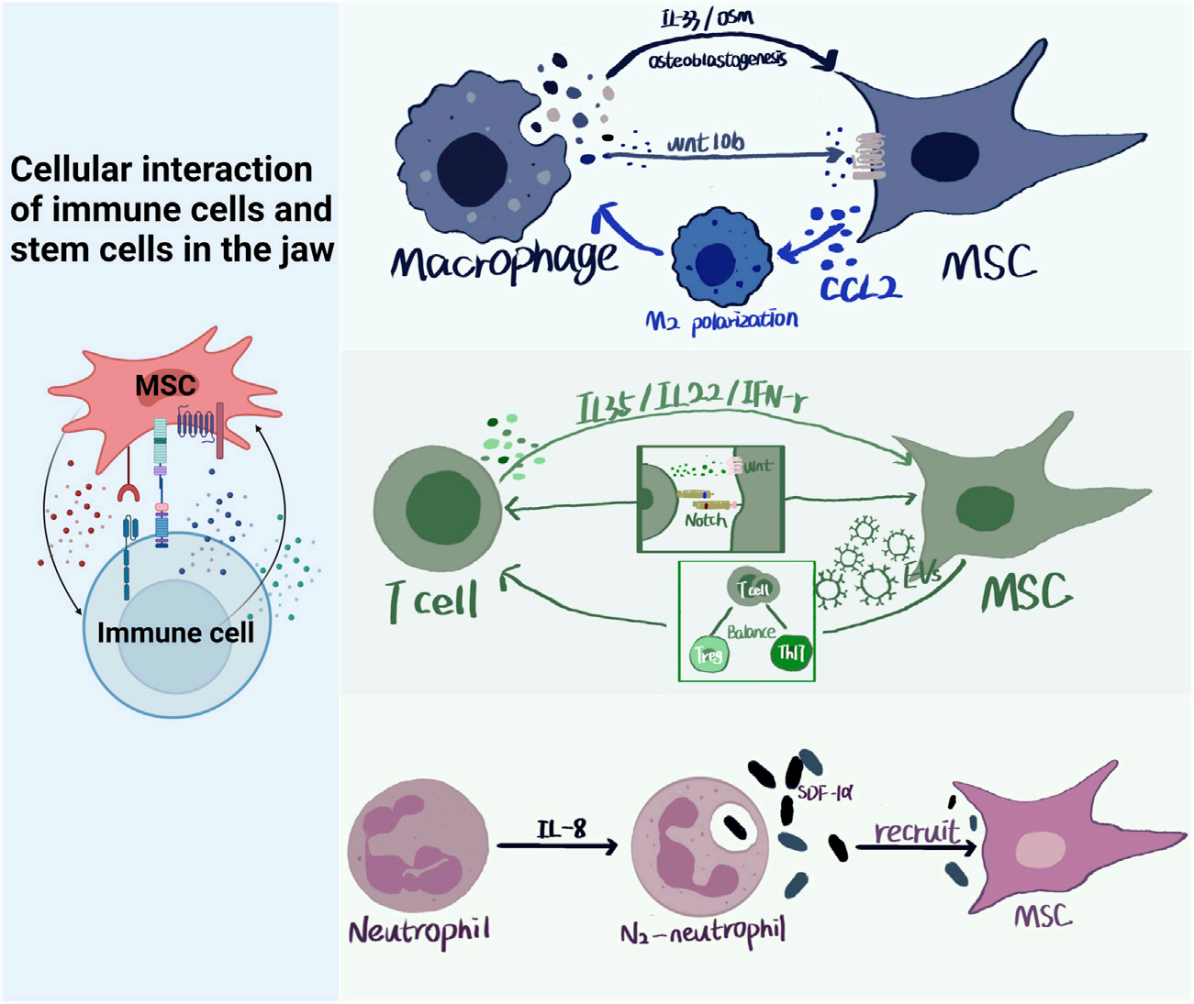
Cellular interaction of immune cells and stem cells in the jaw. Immune cells and MSCs in the jaw have multiple modes of action, paracrine, signaling pathways, ligand and receptor binding, etc. Immune cells produce IL-35, IL-33, OSM, etc., which act on MSCs through paracrine. MSCs can produce CCL2 to polarize macrophages towards M2 type, and can also produce EVs to regulate Treg/Th17 balance. Neutrophils can recruit MSCs by producing SDF-α.

The interactions between immune cells and jaw stem cells play a crucial role in jaw development, homeostasis, and repair (TSUKASAKI and TAKAYANAGI, 2019). Bone stem cell differentiation is influenced by changes in the immune environment, leading to osteogenic or osteoclast differentiation (Figure 5).

### 5.1 Interactions of immune cells and stem cells in jaw development

Jaw development is inseparable from the immune and stem cells, and damage to either side leads to abnormal jaw development. During jaw development, immune and bone cells share a microenvironment (OKAMOTO et al., 2017). Jaw development is closely associated with the immune system. Immune cells play a role in tissue remodeling and repair of damaged tissues during development (MEZU-NDUBUISI and MAHESHWARI, 2021). BMSCs support the differentiation of immune cells (CALVI et al., 2003). For example, BMSCs can maintain T cell generation by producing Notch ligands (YU et al., 2015). Previously, we also mentioned that MSCs can affect the polarization of macrophages (ZHANG et al., 2010; YANG et al., 2023). The interaction between jaw immune and stem cells is the cornerstone of normal jaw development.

Autosomal recessive osteopetrosis is a disease of abnormal bone development involving the mandible (GARCíA et al., 2013; SAĞLAM et al., 2017). Osteoclast dysfunction in patients with this disease (PENNA et al., 2021). Osteoclasts are derive from monocyte/macrophage-lineage cells, which is conducive to the catabolism of jawbone (MCDONALD et al., 2021). Jacome-Galarza et al. found that timely infusion of monocytes into neonatal mice can save bone dysplasia caused by early autosomal bone hyperplasia (JACOME-GALARZA et al., 2019). It may also be possible to treat the disease by modifying the existing immune environment. One study has found that T cell subsets like Th1 and Th17 help promote osteoclast production (LI J. et al., 2020). TNF-α, IL-1β and IL-6 produced by immune cells also increase osteoclast production (RANA et al., 2018).

Gaucher disease (GD) is an autosomal recessive disease associated with jaw bone involvement (MOHAMED et al., 2020). Patients with GD, mainly macrophages, have impaired immune cell functions, resulting in unbalanced bone formation and breakdown (STIRNEMANN et al., 2017). The damaged macrophages produced numerous pro-inflammatory factors, including IL-1β, TNF-α, and IL-6, which act on MSCs through paracrine, resulting in the inhibition of osteogenic differentiation of MSCs (MUCCI and ROZENFELD, 2015; YAO et al., 2021). The severity of the disease in GD have a positive correlation with the levels of pro-inflammatory cytokines such as IL-1β, TNF-α, and M-CSF (CHEN et al., 2018). Therefore, a new treatment scheme for bone dysplasia caused by GD can be provided by down-regulating the level of proinflammatory factors.

### 5.2 Interactions of immune cells and stem cells during jaw homeostasis

Normal bone homeostasis is necessary to maintain jaw health. Bone homeostasis is maintained through a balance between osteoblasts and osteoclasts, and the immune system has an important influence on bone homeostasis (SALHOTRA et al., 2020). The uniqueness of jaw immune environment is also an important factor to help maintain jaw homeostasis, such as higher proportion of macrophages (LIN et al., 2021). Dysregulated jaw homeostasis generally leads to bone disease.

Periodontitis is a disease in which jaw homeostasis is disrupted, often resulting in bone loss. Modulation of M1/M2 macrophage phenotypes can slow inflammation and reduce bone loss in periodontitis (HUANG et al., 2022). Macrophage macrophages promote osteogenic differentiation of MSCs by secreting OSM and BMP-2 (ZHANG Y. et al., 2017). OSM also can increasing the number of M2-type macrophages to help relieve bone loss from periodontitis (YUAN et al., 2023). Tregs inhibit the absorption of alveolar bone by secreting TGF-β and IL-10 (CAFFERATA et al., 2020). The interaction between Tregs and CD8+T cells increases Wnt10b secretion, thereby regulating bone anabolism (TYAGI et al., 2018). Maintenance of jaw homeostasis in periodontitis is also related to other immune cells, such as B cells, neutrophils, and mast cells. Rational use of immune response is a common method in clinical treatment of periodontitis.

Heterotopic ossification (HO) refers to the formation of extraskeletal bone in none-osseous tissues. Macrophages are key cells involved in HO (AGARWAL et al., 2016). Macrophages express high levels of osteogenic factors, including OSM and BMP, resulting in excessive osteogenic differentiation of BMSCs (HUANG et al., 2021). TGF-β produced by immune cells in HO exacerbates the disease, by inducing MSC migration and osteogenic differentiation (MAO et al., 2020). Recent studies have shown that neutrophils are involved in HO injury and that toll-like receptor signaling is highly expressed in cells at the site of injury (NUNEZ et al., 2023). Toll-like receptor signaling regulates BMSC function (NEMETH et al., 2010). Inhibiting the effection of immune cells on MSC osteogenic differentiation can be an effective means to inhibit HO.

### 5.3 Stem cells in appropriate immune microenvironment promote injury repair of jaw

During the healing process of jaw injury, different immune cell compositions and activation energies in the immune cell environment have favorable effects on jaw injury repair (MUIRE et al., 2020). The repair function of MSCs in inflammatory diseases depends on their immune microenvironment (XIONG et al., 2022). Here, we list several clinical jaw injury diseases and review the correlation between the immune microenvironment and jaw stem cells, from a pathological perspective, to promote jaw defect repair.

Jaw fractures are common traumatic jaw diseases. Fracture healing begins with an inflammatory response and the immune system is involved in the healing process (CLAES et al., 2012). Early in a fracture, neutrophils are recruited to the injury site (BASTIAN et al., 2011). Under inflammatory conditions, neutrophils produce chemokines that recruit BMSCs (CAI et al., 2021). The recruited MSCs can induce the production of Tregs, which can promote fracture healing by reducing the levels of IFN-γ and TNF-α (LIU et al., 2011). Macrophages also play a key role in wound healing. In the early stage of fracture, M1 macrophages can stimulate inflammation, and in the later stage, M2 macrophages help fracture healing by producing OSM, PGE2 and BMP-2 (PAJARINEN et al., 2019). Moreover, Macrophages directly promotes the MSC osteogenic differentiation to aid fracture healing (ZHANG et al., 2022).

Alveolar bone regeneration is a key step in restoring healthy jaw function after tooth extraction. Immediately after tooth extraction, neutrophils are recruited to activate other immune cells (COSKUN BENLIDAYI and GUZEL, 2013). Subsequently, macrophages are recruited and mature. When the aggregation of macrophages is inhibited, the healing of alveolar bone slows down (AOYAGI et al., 2018). Reducing the number of lymphocytes and eosinophils in tooth extraction patients is significantly associated with delayed wound healing (HAYASHI et al., 2018). Therefore, a suitable immune environment is necessary for alveolar bone healing after tooth extraction.

In conclusion, the interaction between jaw stem cells and immune cells is very complex and runs through the process of jaw development, maintenance and injury repair. However, the current research is limited, additional studies are warranted to elucidate the potential mechanisms of interaction.

## 6 Conclusion and outlook

In recent years, an increasing number of studies have investigated the correlation between stem cells and immune cells. The jawbone has attracted considerable attention owing to its unique immune environment.

Bone loss is associated with osteoblast balance disruption, decreased osteoblast numbers, and increased osteoclast proliferation. A significant accumulation of immune cells and MSCs occurs at the bone resorption site, leading to a cascade of reactions. Immune cells located in the jaw can enhance the osteogenic differentiation of jaw stem cells and facilitate the repair of jaw defects through the simultaneous secretion of various substances and activation of many signaling pathways. Stem cells in the jawbone can exert immunomodulatory effects and affect the immune microenvironment, thereby helping regenerate the jawbone. We hope that this review will provide valuable insights into jaw loss treatment.

## References

[B1] Abaricia J. O. Farzad N. Heath T. J. Simmons J. Morandini L. Olivares-Navarrete R. (2021). Control of innate immune response by biomaterial surface topography, energy, and stiffness. Acta biomater. 133, 58–73. 10.1016/j.actbio.2021.04.021 33882355 PMC8497396

[B2] Agarwal S. Loder S. Brownley C. Cholok D. Mangiavini L. Li J. (2016). Inhibition of Hif1α prevents both trauma-induced and genetic heterotopic ossification. Proc. Natl. Acad. Sci. U. S. A. 113 (3), E338–E347. 10.1073/pnas.1515397113 26721400 PMC4725488

[B3] Al-Azab M. Walana W. Wei J. Li W. Tang Y. Wei X. (2021). TL1A/TNFR2 Axis enhances immunoregulatory effects of bone marrow derived mesenchymal stem cell by Indian hedgehog signaling pathway. Int. J. stem cells 14 (1), 58–73. 10.15283/ijsc19121 33122466 PMC7904531

[B4] Alvarez-Medina R. Cayuso J. Okubo T. Takada S. Martí E. (2008). Wnt canonical pathway restricts graded Shh/Gli patterning activity through the regulation of Gli3 expression. Dev. Camb. Engl. 135 (2), 237–247. 10.1242/dev.012054 18057099

[B5] Amarasekara D. S. Kim S. Rho J. (2021). Regulation of osteoblast differentiation by cytokine networks. Int. J. Mol. Sci. 22 (6), 2851. 10.3390/ijms22062851 33799644 PMC7998677

[B6] Aoyagi H. Yamashiro K. Hirata-Yoshihara C. Ideguchi H. Yamasaki M. Kawamura M. (2018). HMGB1-induced inflammatory response promotes bone healing in murine tooth extraction socket. J. Cell. Biochem. 119 (7), 5481–5490. 10.1002/jcb.26710 29377249

[B7] Ashour L. Al Habashneh R. A. Al-Mrahelh M. M. Abuarqoub D. Khader Y. S. Jafar H. (2020). The modulation of mature dendritic cells from patients with type 1 diabetes using human periodontal ligament stem cells. An *in-vitro* study. J. diabetes metabolic Disord. 19 (2), 1037–1044. 10.1007/s40200-020-00602-4 PMC7843723 33520821

[B8] Bastian O. Pillay J. Alblas J. Leenen L. Koenderman L. Blokhuis T. (2011). Systemic inflammation and fracture healing. J. Leukoc. Biol. 89 (5), 669–673. 10.1189/jlb.0810446 21208896

[B9] Bi R. Yin Q. Li H. Yang X. Wang Y. (2023). A single-cell transcriptional atlas reveals resident progenitor cell niche functions in TMJ disc development and injury. Nat. Commun. 14 (1), 830. 10.1038/s41467-023-36406-2 36788226 PMC9929076

[B10] Cafferata E. A. Terraza-Aguirre C. Barrera R. Faúndez N. González N. Rojas C. (2020). Interleukin-35 inhibits alveolar bone resorption by modulating the Th17/Treg imbalance during periodontitis. J. Clin. periodontology 47 (6), 676–688. 10.1111/jcpe.13282 32160331

[B11] Cai B. Lin D. Li Y. Wang L. Xie J. Dai T. (2021). N2-Polarized neutrophils guide bone mesenchymal stem cell recruitment and initiate bone regeneration: a missing piece of the bone regeneration puzzle. Adv. Sci. 8 (19), e2100584. 10.1002/advs.202100584 PMC8498914 34382372

[B12] Cai G. Lu Y. Zhong W. Wang T. Li Y. Ruan X. (2023). Piezo1-mediated M2 macrophage mechanotransduction enhances bone formation through secretion and activation of transforming growth factor-β1. Cell Prolif. 56, e13440. 10.1111/cpr.13440 36880296 PMC10472522

[B13] Calvi L. M. Adams G. B. Weibrecht K. W. Weber J. M. Olson D. P. Knight M. C. (2003). Osteoblastic cells regulate the haematopoietic stem cell niche. Nature 425 (6960), 841–846. 10.1038/nature02040 14574413

[B14] Cao F. Zhan J. Chen X. Zhang K. Lai R. Feng Z. (2017). miR-214 promotes periodontal ligament stem cell osteoblastic differentiation by modulating Wnt/β‑catenin signaling. Mol. Med. Rep. 16 (6), 9301–9308. 10.3892/mmr.2017.7821 29152645 PMC5779983

[B15] Cao G. Zhang X. Song Y. Sun Y. Ling H. Han X. (2021). Local promotion of B10 function alleviates experimental periodontitis bone loss through antagonizing RANKL-expressing neutrophils. J. periodontology 92 (6), 907–920. 10.1002/JPER.20-0074 32845513

[B16] Carr M. J. Toma J. S. Johnston A. P. W. Steadman P. E. Yuzwa S. A. Mahmud N. (2019). Mesenchymal precursor cells in adult nerves contribute to mammalian tissue repair and regeneration. Cell stem Cell 24 (2), 240–256. 10.1016/j.stem.2018.10.024 30503141

[B17] Cassatella M. A. Mosna F. Micheletti A. Lisi V. Tamassia N. Cont C. (2011). Toll-like receptor-3-activated human mesenchymal stromal cells significantly prolong the survival and function of neutrophils. Stem cells Dayt. Ohio 29 (6), 1001–1011. 10.1002/stem.651 21563279

[B18] Chakraborty M. Chu K. Shrestha A. Revelo X. S. Zhang X. Gold M. J. (2021). Mechanical stiffness controls dendritic cell metabolism and function. Cell Rep. 34 (2), 108609. 10.1016/j.celrep.2020.108609 33440149

[B19] Cheat B. Torrens C. Foda A. Baroukh B. Sadoine J. Slimani L. (2022). NLRP3 is involved in neutrophil mobilization in experimental periodontitis. Front. Immunol. 13, 839929. 10.3389/fimmu.2022.839929 35281020 PMC8905524

[B20] Chen X. Wan Z. Yang L. Song S. Fu Z. Tang K. (2022). Exosomes derived from reparative M2-like macrophages prevent bone loss in murine periodontitis models via IL-10 mRNA. J. nanobiotechnology 20 (1), 110. 10.1186/s12951-022-01314-y 35248085 PMC8898524

[B21] Chen Y. Sud N. Hettinghouse A. Liu C. J. (2018). Molecular regulations and therapeutic targets of Gaucher disease. Cytokine and growth factor Rev. 41, 65–74. 10.1016/j.cytogfr.2018.04.003 29699937 PMC8108120

[B22] Chen Y. Wang H. Ni Q. Wang T. Bao C. Geng Y. (2023). B-Cell-Derived TGF-β1 inhibits osteogenesis and contributes to bone loss in periodontitis. J. Dent. Res. 102 (7), 767–776. 10.1177/00220345231161005 37082865

[B23] Claes L. Recknagel S. Ignatius A. (2012). Fracture healing under healthy and inflammatory conditions. Nat. Rev. Rheumatol. 8 (3), 133–143. 10.1038/nrrheum.2012.1 22293759

[B24] Coskun Benlidayi I. Guzel R. (2013). Oral bisphosphonate related osteonecrosis of the jaw: a challenging adverse effect. ISRN Rheumatol. 2013, 215034. 10.1155/2013/215034 23762600 PMC3671545

[B25] Elashiry M. Elashiry M. M. Elsayed R. Rajendran M. Auersvald C. Zeitoun R. (2020). Dendritic cell derived exosomes loaded with immunoregulatory cargo reprogram local immune responses and inhibit degenerative bone disease *in vivo* . J. Extracell. vesicles 9 (1), 1795362. 10.1080/20013078.2020.1795362 32944183 PMC7480413

[B26] El-Awady A. R. Elashiry M. Morandini A. C. Meghil M. M. Cutler C. W. (2022). Dendritic cells a critical link to alveolar bone loss and systemic disease risk in periodontitis: immunotherapeutic implications. Periodontology 89 (1), 41–50. 10.1111/prd.12428 PMC9018591 35244951

[B27] Elsayed R. Kurago Z. Cutler C. W. Arce R. M. Gerber J. Celis E. (2020). Role of dendritic cell-mediated immune response in oral homeostasis: a new mechanism of osteonecrosis of the jaw. FASEB J. 34 (2), 2595–2608. 10.1096/fj.201901819RR 31919918 PMC7712496

[B28] Gao Y. Duan R. Li H. Jiang L. Tao T. Liu X. (2023). Single-cell analysis of immune cells on gingiva-derived mesenchymal stem cells in experimental autoimmune uveitis. iScience 26 (5), 106729. 10.1016/j.isci.2023.106729 37216113 PMC10192653

[B29] GarcíA C. M. GarcíA M. A. GarcíA R. G. Gil F. M. (2013). Osteomyelitis of the mandible in a patient with osteopetrosis. Case report and review of the literature. J. Maxillofac. oral Surg. 12 (1), 94–99. 10.1007/s12663-011-0196-y 24431821 PMC3589508

[B30] Ghorpade D. S. Holla S. Kaveri S. V. Bayry J. Patil S. A. Balaji K. N. (2013). Sonic hedgehog-dependent induction of microRNA 31 and microRNA 150 regulates Mycobacterium bovis BCG-driven toll-like receptor 2 signaling. Mol. Cell. Biol. 33 (3), 543–556. 10.1128/MCB.01108-12 23166298 PMC3666883

[B31] Gong X. Zhang H. Xu X. Ding Y. Yang X. Cheng Z. (2022). Tracing PRX1(+) cells during molar formation and periodontal ligament reconstruction. Int. J. Oral Sci. 14 (1), 5. 10.1038/s41368-021-00155-z 35078971 PMC8789835

[B32] Guan C. C. Yan M. Jiang X. Q. Zhang P. Zhang X. L. Li J. (2009). Sonic hedgehog alleviates the inhibitory effects of high glucose on the osteoblastic differentiation of bone marrow stromal cells. Bone 45 (6), 1146–1152. 10.1016/j.bone.2009.08.009 19683085

[B33] Guiglia R. Di Fede O. Lo Russo L. Sprini D. Rini G. B. Campisi G. (2013). Osteoporosis, jawbones and periodontal disease. Patol. oral cirugia bucal 18 (1), e93–e99. 10.4317/medoral.18298 PMC3548653 23229255

[B34] Ha D. H. Kim H. K. Lee J. Kwon H. H. Park G. H. Yang S. H. (2020). Mesenchymal stem/stromal cell-derived exosomes for immunomodulatory therapeutics and skin regeneration. Cells 9 (5), 1157. 10.3390/cells9051157 32392899 PMC7290908

[B35] Hajishengallis G. Moutsopoulos N. M. Hajishengallis E. Chavakis T. (2016). Immune and regulatory functions of neutrophils in inflammatory bone loss. Seminars Immunol. 28 (2), 146–158. 10.1016/j.smim.2016.02.002 PMC4867283 26936034

[B36] Halloran D. Durbano H. W. Nohe A. (2020). Bone morphogenetic protein-2 in development and bone homeostasis. J. Dev. Biol. 8 (3), 19. 10.3390/jdb8030019 32933207 PMC7557435

[B37] Han Y. Jin Y. Miao Y. Shi T. Lin X. (2018). Switched memory B cells promote alveolar bone damage during periodontitis: an adoptive transfer experiment. Int. Immunopharmacol. 62, 147–154. 10.1016/j.intimp.2018.07.003 30015235

[B38] Hayashi M. Morimoto Y. Iida T. Tanaka Y. Sugiyama S. (2018). Risk of delayed healing of tooth extraction wounds and osteonecrosis of the jaw among patients treated with potential immunosuppressive drugs: a retrospective cohort study. Tohoku J. Exp. Med. 246 (4), 257–264. 10.1620/tjem.246.257 30568073

[B39] He X. Dong Z. Cao Y. Wang H. Liu S. Liao L. (2019). MSC-derived exosome promotes M2 polarization and enhances cutaneous wound healing. Stem cells Int. 2019, 7132708. 10.1155/2019/7132708 31582986 PMC6754952

[B40] He X. Jiang W. Luo Z. Qu T. Wang Z. Liu N. (2017). IFN-γ regulates human dental pulp stem cells behavior via NF-κB and MAPK signaling. Sci. Rep. 7, 40681. 10.1038/srep40681 28098169 PMC5241669

[B41] Hosoya A. Shalehin N. Takebe H. Fujii S. Seki Y. Mizoguchi T. (2020). Stem cell properties of Gli1-positive cells in the periodontal ligament. J. oral Biosci. 62 (4), 299–305. 10.1016/j.job.2020.08.002 32882366

[B42] Hovav A. H. (2014). Dendritic cells of the oral mucosa. Mucosal Immunol. 7 (1), 27–37. 10.1038/mi.2013.42 23757304

[B43] Huang H. Pan W. Wang Y. Kim H. S. Shao D. Huang B. (2022). Nanoparticulate cell-free DNA scavenger for treating inflammatory bone loss in periodontitis. Nat. Commun. 13 (1), 5925. 10.1038/s41467-022-33492-6 36207325 PMC9546917

[B44] Huang X. Cheng B. Song W. Wang L. Zhang Y. Hou Y. (2020). Superior CKIP-1 sensitivity of orofacial bone-derived mesenchymal stem cells in proliferation and osteogenic differentiation compared to long bone-derived mesenchymal stem cells. Mol. Med. Rep. 22 (2), 1169–1178. 10.3892/mmr.2020.11239 32626993 PMC7339610

[B45] Huang Y. Wang X. Zhou D. Zhou W. Dai F. Lin H. (2021). Macrophages in heterotopic ossification: from mechanisms to therapy. NPJ Regen. Med. 6 (1), 70. 10.1038/s41536-021-00178-4 34702860 PMC8548514

[B46] Hu H. Hilton M. J. Tu X. Yu K. Ornitz D. M. Long F. (2005). Sequential roles of Hedgehog and Wnt signaling in osteoblast development. Development 132 (1), 49–60. 10.1242/dev.01564 15576404

[B47] Hu Y. Tian H. Chen W. Liu Y. Cao Y. Pei H. (2023). The critical role of the piezo1/β-catenin/ATF4 Axis on the stemness of Gli1(+) BMSCs during simulated microgravity-induced bone loss. Adv. Sci. Weinheim, Baden-Wurttemberg, Ger. 10 (32), e2303375. 10.1002/advs.202303375 PMC10646271 37759400

[B48] Imanishi Y. Hata M. Matsukawa R. Aoyagi A. Omi M. Mizutani M. (2021). Efficacy of extracellular vesicles from dental pulp stem cells for bone regeneration in rat calvarial bone defects. . Inflamm. Regen. 41 (1), 12. 10.1186/s41232-021-00163-w 33853679 PMC8048358

[B49] Itoyama T. Yoshida S. Tomokiyo A. Hasegawa D. Hamano S. Sugii H. (2020). Possible function of GDNF and Schwann cells in wound healing of periodontal tissue. J. periodontal Res. 55 (6), 830–839. 10.1111/jre.12774 32562261

[B50] Jacome-Galarza C. E. Percin G. I. Muller J. T. Mass E. Lazarov T. Eitler J. (2019). Developmental origin, functional maintenance and genetic rescue of osteoclasts. Nature 568 (7753), 541–545. 10.1038/s41586-019-1105-7 30971820 PMC6910203

[B51] Jeyaraman M. Verma T. Jeyaraman N. Patro B. P. Nallakumarasamy A. Khanna M. (2023). Is mandible derived mesenchymal stromal cells superior in proliferation and regeneration to long bone-derived mesenchymal stromal cells? World J. Methodol. 13 (2), 10–17. 10.5662/wjm.v13.i2.10 37035028 PMC10080497

[B52] Jiang S. Xu L. (2020). Exosomes from gingival mesenchymal stem cells enhance migration and osteogenic differentiation of pre-osteoblasts. Die Pharm. 75 (11), 576–580. 10.1691/ph.2020.0652 33239132

[B53] Jin A. Xu H. Gao X. Sun S. Yang Y. Huang X. (2023). ScRNA-seq reveals a distinct osteogenic progenitor of alveolar bone. J. Dent. Res. 102 (6), 645–655. 10.1177/00220345231159821 37148259

[B54] Jones R. E. Salhotra A. Robertson K. S. Ransom R. C. Foster D. S. Shah H. N. (2019). Skeletal stem cell-schwann cell circuitry in mandibular repair. Cell Rep. 28 (11), 2757–2766. 10.1016/j.celrep.2019.08.021 31509739 PMC6820052

[B55] Kang M. Huang C. C. Lu Y. Shirazi S. Gajendrareddy P. Ravindran S. (2020). Bone regeneration is mediated by macrophage extracellular vesicles. Bone 141, 115627. 10.1016/j.bone.2020.115627 32891867 PMC8107826

[B56] Kaukua N. Shahidi M. K. Konstantinidou C. Dyachuk V. Kaucka M. Furlan A. (2014). Glial origin of mesenchymal stem cells in a tooth model system. Nature 513 (7519), 551–554. 10.1038/nature13536 25079316

[B57] Kitamura A. Kawasaki M. Kawasaki K. Meguro F. Yamada A. Nagai T. (2020). Ift88 is involved in mandibular development. J. Anat. 236 (2), 317–324. 10.1111/joa.13096 31657471 PMC6956436

[B58] KöNNECKE I. Serra A. El Khassawna T. Schlundt C. Schell H. Hauser A. (2014). T and B cells participate in bone repair by infiltrating the fracture callus in a two-wave fashion. Bone 64, 155–165. 10.1016/j.bone.2014.03.052 24721700

[B59] Koyama N. Okubo Y. Nakao K. Osawa K. Fujimura K. Bessho K. (2011). Pluripotency of mesenchymal cells derived from synovial fluid in patients with temporomandibular joint disorder. Life Sci. 89 (19-20), 741–747. 10.1016/j.lfs.2011.09.005 21958469

[B60] Kunimatsu R. Nakajima K. Awada T. Tsuka Y. Abe T. Ando K. (2018). Comparative characterization of stem cells from human exfoliated deciduous teeth, dental pulp, and bone marrow-derived mesenchymal stem cells. Biochem. biophysical Res. Commun. 501 (1), 193–198. 10.1016/j.bbrc.2018.04.213 29730288

[B61] Kwack K. H. Lamb N. A. Bard J. E. Kramer E. D. Zhang L. Abrams S. I. (2021). Discovering myeloid cell heterogeneity in mandibular bone - cell by cell analysis. Front. physiology 12, 731549. 10.3389/fphys.2021.731549 PMC8514701 34658914

[B62] Lee A. E. Choi J. G. Shi S. H. He P. Zhang Q. Z. Le A. D. (2023). DPSC-derived extracellular vesicles promote rat jawbone regeneration. J. Dent. Res. 102 (3), 313–321. 10.1177/00220345221133716 36348514

[B63] Lee Y. C. Chan Y. H. Hsieh S. C. Lew W. Z. Feng S. W. (2019). Comparing the osteogenic potentials and bone regeneration capacities of bone marrow and dental pulp mesenchymal stem cells in a rabbit calvarial bone defect model. Int. J. Mol. Sci. 20 (20), 5015. 10.3390/ijms20205015 31658685 PMC6834129

[B64] Leng S. Xu W. Wu L. Liu L. Du J. Yang F. (2023). NLRP3 disturbs Treg/Th17 cell balance to aggravate apical periodontitis. J. Dent. Res. 102 (6), 656–666. 10.1177/00220345231151692 36883625

[B65] Liao C. Zhang C. Jin L. Yang Y. (2020). IL-17 alters the mesenchymal stem cell niche towards osteogenesis in cooperation with osteocytes. J. Cell. physiology 235 (5), 4466–4480. 10.1002/jcp.29323 PMC7113695 31643095

[B66] Liew P. X. Kubes P. (2019). The neutrophil's role during health and disease. Physiol. Rev. 99 (2), 1223–1248. 10.1152/physrev.00012.2018 30758246

[B67] Li J. Tan J. Martino M. M. Lui K. O. (2018). Regulatory T-cells: potential regulator of tissue repair and regeneration. Front. Immunol. 9, 585. 10.3389/fimmu.2018.00585 29662491 PMC5890151

[B68] Li J. Yu T. T. Yan H. C. Qiao Y. Q. Wang L. C. Zhang T. (2020b). T cells participate in bone remodeling during the rapid palatal expansion. FASEB J. 34 (11), 15327–15337. 10.1096/fj.202001078R 32951236

[B69] Lin W. M. Yuan Q. (2022). Latest research findings on immune microenvironment regulation in jawbone-related diseases. J. Sichuan Univ. Med. Sci. Ed. 53 (3), 528–531. 10.12182/20220560502 PMC10409418 35642166

[B70] Lin T. Pajarinen J. Nabeshima A. Lu L. Nathan K. Jämsen E. (2017). Preconditioning of murine mesenchymal stem cells synergistically enhanced immunomodulation and osteogenesis. Stem Cell Res. Ther. 8 (1), 277. 10.1186/s13287-017-0730-z 29212557 PMC5719931

[B71] Lin W. Li Q. Zhang D. Zhang X. Qi X. Wang Q. (2021). Mapping the immune microenvironment for mandibular alveolar bone homeostasis at single-cell resolution. Bone Res. 9 (1), 17. 10.1038/s41413-021-00141-5 33723232 PMC7960742

[B72] Li P. Ou Q. Shi S. Shao C. (2023a). Immunomodulatory properties of mesenchymal stem cells/dental stem cells and their therapeutic applications. Cell. Mol. Immunol. 20 (6), 558–569. 10.1038/s41423-023-00998-y 36973490 PMC10040934

[B73] Li Q. Yang G. Li J. Ding M. Zhou N. Dong H. (2020a). Stem cell therapies for periodontal tissue regeneration: a network meta-analysis of preclinical studies. Stem Cell Res. Ther. 11 (1), 427. 10.1186/s13287-020-01938-7 33008471 PMC7531120

[B74] Li S. Su L. Luan Q. Liu G. Zeng W. Yu X. (2023b). Regulatory B cells induced by interleukin-35 inhibit inflammation and alveolar bone resorption in ligature-induced periodontitis. J. periodontology 94, 1376–1388. 10.1002/JPER.23-0038 37086023

[B75] Liu A. Jin S. Fu C. Cui S. Zhang T. Zhu L. (2020). Macrophage-derived small extracellular vesicles promote biomimetic mineralized collagen-mediated endogenous bone regeneration. Int. J. Oral Sci. 12 (1), 33. 10.1038/s41368-020-00100-6 33257654 PMC7705747

[B76] Liu J. Chen B. Bao J. Zhang Y. Lei L. Yan F. (2019). Macrophage polarization in periodontal ligament stem cells enhanced periodontal regeneration. Stem Cell Res. Ther. 10 (1), 320. 10.1186/s13287-019-1409-4 31730019 PMC6858751

[B77] Liu L. Guo S. Shi W. Liu Q. Huo F. Wu Y. (2021). Bone marrow mesenchymal stem cell-derived small extracellular vesicles promote periodontal regeneration. Tissue Eng. Part A 27 (13-14), 962–976. 10.1089/ten.TEA.2020.0141 32962564

[B78] Liu Y. Wang L. Kikuiri T. Akiyama K. Chen C. Xu X. (2011). Mesenchymal stem cell-based tissue regeneration is governed by recipient T lymphocytes via IFN-γ and TNF-α. Nat. Med. 17 (12), 1594–1601. 10.1038/nm.2542 22101767 PMC3233650

[B79] Li W. Wang C. Wang Z. Gou L. Zhou Y. Peng G. (2022). Physically cross-linked DNA hydrogel-based sustained cytokine delivery for *in situ* diabetic alveolar bone rebuilding. ACS Appl. Mater. interfaces 14 (22), 25173–25182. 10.1021/acsami.2c04769 35638566

[B80] Li X. Han L. Nookaew I. Mannen E. Silva M. J. Almeida M. (2019). Stimulation of Piezo1 by mechanical signals promotes bone anabolism. eLife 8, e49631. 10.7554/eLife.49631 31588901 PMC6779475

[B81] Li Y. Ling J. Jiang Q. (2021b). Inflammasomes in alveolar bone loss. Front. Immunol. 12, 691013. 10.3389/fimmu.2021.691013 34177950 PMC8221428

[B82] Li Z. Wang Y. Li S. Li Y. (2021a). Exosomes derived from M2 macrophages facilitate osteogenesis and reduce adipogenesis of BMSCs. Front. Endocrinol. 12, 680328. 10.3389/fendo.2021.680328 PMC8290518 34295306

[B83] Lopes D. Martins-Cruz C. Oliveira M. B. Mano J. F. (2018). Bone physiology as inspiration for tissue regenerative therapies. Biomaterials 185, 240–275. 10.1016/j.biomaterials.2018.09.028 30261426 PMC6445367

[B84] Lopez-Leal R. Court F. A. (2016). Schwann cell exosomes mediate neuron-glia communication and enhance axonal regeneration. Cell. Mol. Neurobiol. 36 (3), 429–436. 10.1007/s10571-015-0314-3 26993502 PMC11482438

[B85] Luo X. Wan Q. Cheng L. Xu R. (2022). Mechanisms of bone remodeling and therapeutic strategies in chronic apical periodontitis. Front. Cell. Infect. Microbiol. 12, 908859. 10.3389/fcimb.2022.908859 35937695 PMC9353524

[B86] Mahon O. R. Browe D. C. Gonzalez-Fernandez T. Pitacco P. Whelan I. T. Von Euw S. (2020). Nano-particle mediated M2 macrophage polarization enhances bone formation and MSC osteogenesis in an IL-10 dependent manner. Biomaterials 239, 119833. 10.1016/j.biomaterials.2020.119833 32062479

[B87] Ma L. Hu J. Cao Y. Xie Y. Wang H. Fan Z. (2019). Maintained properties of aged dental pulp stem cells for superior periodontal tissue regeneration. Aging Dis. 10 (4), 793–806. 10.14336/AD.2018.0729 31440385 PMC6675537

[B88] Mao D. Pan X. Rui Y. Li F. (2020). Matrine attenuates heterotopic ossification by suppressing TGF-β induced mesenchymal stromal cell migration and osteogenic differentiation. Biomed. Pharmacother. 127, 110152. 10.1016/j.biopha.2020.110152 32559842

[B89] Maruhashi T. Kaifu T. Yabe R. Seno A. Chung S. H. Fujikado N. (2015). DCIR maintains bone homeostasis by regulating IFN-γ production in T cells. J. Immunol. 194 (12), 5681–5691. 10.4049/jimmunol.1500273 25926676

[B90] Maruyama T. (2019). Stem cells of the suture mesenchyme in craniofacial bone development, repair and regeneration. Keio J. Med. 68 (2), 42. 10.2302/kjm.68-003-ABST 31243185

[B91] Matsumura-Kawashima M. Ogata K. Moriyama M. Murakami Y. Kawado T. Nakamura S. (2021). Secreted factors from dental pulp stem cells improve Sjögren's syndrome via regulatory T cell-mediated immunosuppression. Stem Cell Res. Ther. 12 (1), 182. 10.1186/s13287-021-02236-6 33726818 PMC7962357

[B92] Mcdonald M. M. Khoo W. H. Ng P. Y. Xiao Y. Zamerli J. Thatcher P. (2021). Osteoclasts recycle via osteomorphs during RANKL-stimulated bone resorption. Cell 184 (5), 1940–1947.e13. 10.1016/j.cell.2021.03.010 33798441 PMC8024244

[B93] Men Y. Wang Y. Yi Y. Jing D. Luo W. Shen B. (2020). Gli1+ periodontium stem cells are regulated by osteocytes and occlusal force. Dev. Cell 54 (5), 639–654. 10.1016/j.devcel.2020.06.006 32652075

[B94] Mezu-Ndubuisi O. J. Maheshwari A. (2021). Role of macrophages in fetal development and perinatal disorders. Pediatr. Res. 90 (3), 513–523. 10.1038/s41390-020-01209-4 33070164

[B95] Mohamed Y. S. A. Zayet M. K. Omar O. M. El-Beshlawy A. M. (2020). Jaw bones' involvement and dental features of type I and type III Gaucher disease: a radiographic study of 42 paediatric patients. official J. Eur. Acad. Paediatr. Dent. 21 (2), 241–247. 10.1007/s40368-019-00471-3 31531808

[B96] Mucci J. M. Rozenfeld P. (2015). Pathogenesis of bone alterations in gaucher disease: the role of immune system. J. Immunol. Res. 2015, 1–6. 10.1155/2015/192761 PMC4433682 26064996

[B97] Muire P. J. Mangum L. H. Wenke J. C. (2020). Time course of immune response and immunomodulation during normal and delayed healing of musculoskeletal wounds. Front. Immunol. 11, 1056. 10.3389/fimmu.2020.01056 32582170 PMC7287024

[B98] Nakajima R. Ono M. Hara E. S. Oida Y. Shinkawa S. Pham H. T. (2014). Mesenchymal stem/progenitor cell isolation from tooth extraction sockets. J. Dent. Res. 93 (11), 1133–1140. 10.1177/0022034514549377 25170030 PMC4293764

[B99] Nemeth K. Mayer B. Mezey E. (2010). Modulation of bone marrow stromal cell functions in infectious diseases by toll-like receptor ligands. J. Mol. Med. Berlin, Ger. 88 (1), 5–10. 10.1007/s00109-009-0523-7 PMC2928254 19756450

[B100] Nunez J. H. Juan C. Sun Y. Hong J. Bancroft A. C. Hwang C. (2023). Neutrophil and NETosis modulation in traumatic heterotopic ossification. Ann. Surg. 278 (6), e1289–e1298. 10.1097/SLA.0000000000005940 37325925 PMC10724380

[B101] Oka K. Oka S. Sasaki T. Ito Y. Bringas P. Nonaka K. (2007). The role of TGF-beta signaling in regulating chondrogenesis and osteogenesis during mandibular development. Dev. Biol. 303 (1), 391–404. 10.1016/j.ydbio.2006.11.025 17204263 PMC2074881

[B102] Okamoto K. Nakashima T. Shinohara M. Negishi-Koga T. Komatsu N. Terashima A. (2017). Osteoimmunology: the conceptual framework unifying the immune and skeletal systems. Physiol. Rev. 97 (4), 1295–1349. 10.1152/physrev.00036.2016 28814613

[B103] Ono T. Okamoto K. Nakashima T. Nitta T. Hori S. Iwakura Y. (2016). IL-17-producing γδ T cells enhance bone regeneration. Nat. Commun. 7, 10928. 10.1038/ncomms10928 26965320 PMC4792964

[B104] Orsini E. M. Perelas A. Southern B. D. Grove L. M. Olman M. A. Scheraga R. G. (2021). Stretching the function of innate immune cells. Front. Immunol. 12, 767319. 10.3389/fimmu.2021.767319 34795674 PMC8593101

[B105] Pajarinen J. Lin T. Gibon E. Kohno Y. Maruyama M. Nathan K. (2019). Mesenchymal stem cell-macrophage crosstalk and bone healing. Biomaterials 196, 80–89. 10.1016/j.biomaterials.2017.12.025 29329642 PMC6028312

[B106] Peng M. Wang Y. Qiang L. Xu Y. Li C. (2018). Interleukin-35 inhibits TNF-α-induced osteoclastogenesis and promotes apoptosis via shifting the activation from TNF receptor-associated death domain (TRADD)-TRAF2 to TRADD-fas-associated death domain by JAK1/STAT1. Front. Immunol. 9, 1417. 10.3389/fimmu.2018.01417 30061878 PMC6054960

[B107] Penna S. Villa A. Capo V. (2021). Autosomal recessive osteopetrosis: mechanisms and treatments. Dis. models Mech. 14 (5), dmm048940. 10.1242/dmm.048940 PMC8188884 33970241

[B108] Peters S. B. Wang Y. Serra R. (2017). Tgfbr2 is required in osterix expressing cells for postnatal skeletal development. Bone 97, 54–64. 10.1016/j.bone.2016.12.017 28043895 PMC5368008

[B109] Purwaningrum M. Giachelli C. M. Osathanon T. Rattanapuchpong S. Sawangmake C. (2023). Dissecting specific Wnt components governing osteogenic differentiation potential by human periodontal ligament stem cells through interleukin-6. Sci. Rep. 13 (1), 9055. 10.1038/s41598-023-35569-8 37270571 PMC10239497

[B110] Rana A. K. Li Y. Dang Q. Yang F. (2018). Monocytes in rheumatoid arthritis: circulating precursors of macrophages and osteoclasts and, their heterogeneity and plasticity role in RA pathogenesis. Int. Immunopharmacol. 65, 348–359. 10.1016/j.intimp.2018.10.016 30366278

[B111] Redondo L. M. GarcíA V. Peral B. Verrier A. Becerra J. Sánchez A. (2018). Repair of maxillary cystic bone defects with mesenchymal stem cells seeded on a cross-linked serum scaffold. J. cranio-maxillo-facial Surg. 46 (2), 222–229. 10.1016/j.jcms.2017.11.004 29229365

[B112] Redondo-Castro E. Cunningham C. Miller J. Martuscelli L. Aoulad-Ali S. Rothwell N. J. (2017). Interleukin-1 primes human mesenchymal stem cells towards an anti-inflammatory and pro-trophic phenotype *in vitro* . Stem Cell Res. Ther. 8 (1), 79. 10.1186/s13287-017-0531-4 28412968 PMC5393041

[B113] Reichert J. C. Schmalzl J. Prager P. Gilbert F. Quent V. M. C. Steinert A. F. (2013). Synergistic effect of Indian hedgehog and bone morphogenetic protein-2 gene transfer to increase the osteogenic potential of human mesenchymal stem cells. Stem Cell Res. Ther. 4 (5), 105. 10.1186/scrt316 24004723 PMC3854715

[B114] Reis M. Mavin E. Nicholson L. Green K. Dickinson A. M. Wang X. N. (2018). Mesenchymal stromal cell-derived extracellular vesicles attenuate dendritic cell maturation and function. Front. Immunol. 9, 2538. 10.3389/fimmu.2018.02538 30473695 PMC6237916

[B115] Sağlam D. Bilgici M. C. BekçI T. Albayrak C. Albayrak D. (2017). Autosomal recessive osteopetrosis with a unique imaging finding: multiple encephaloceles. Skelet. Radiol. 46 (5), 701–704. 10.1007/s00256-017-2595-8 28233026

[B116] Salhotra A. Shah H. N. Levi B. Longaker M. T. (2020). Mechanisms of bone development and repair. Nat. Rev. Mol. Cell Biol. 21 (11), 696–711. 10.1038/s41580-020-00279-w 32901139 PMC7699981

[B117] Sartoretto S. Gemini-Piperni S. Da Silva R. A. Calasans M. D. Rucci N. Pires Dos Santos T. M. (2019). Apoptosis-associated speck-like protein containing a caspase-1 recruitment domain (ASC) contributes to osteoblast differentiation and osteogenesis. J. Cell. physiology 234 (4), 4140–4153. 10.1002/jcp.27226 30171612

[B118] Shalehin N. Seki Y. Takebe H. Fujii S. Mizoguchi T. Nakamura H. (2022). Gli1(+)-PDL cells contribute to alveolar bone homeostasis and regeneration. J. Dent. Res. 101 (12), 1537–1543. 10.1177/00220345221106921 35786034

[B119] Shapouri-Moghaddam A. Mohammadian S. Vazini H. Taghadosi M. Esmaeili S. A. Mardani F. (2018). Macrophage plasticity, polarization, and function in health and disease. J. Cell. physiology 233 (9), 6425–6440. 10.1002/jcp.26429 PMC13482560 29319160

[B120] Shen Y. Liu C. Yang T. Tang Y. Gu Y. (2023). Transcriptome characterization of human gingival mesenchymal and periodontal ligament stem cells in response to electronic-cigarettes. Environ. Pollut. 323, 121307. 10.1016/j.envpol.2023.121307 36804562

[B121] Shibukawa Y. Young B. Wu C. Yamada S. Long F. Pacifici M. (2007). Temporomandibular joint formation and condyle growth require Indian hedgehog signaling. Dev. Dyn. 236 (2), 426–434. 10.1002/dvdy.21036 17191253

[B122] Shi T. Jin Y. Miao Y. Wang Y. Zhou Y. Lin X. (2020). IL-10 secreting B cells regulate periodontal immune response during periodontitis. Odontology 108 (3), 350–357. 10.1007/s10266-019-00470-2 31701299

[B123] Silva A. M. Almeida M. I. Teixeira J. H. Maia A. F. Calin G. A. Barbosa M. A. (2017). Dendritic cell-derived extracellular vesicles mediate mesenchymal stem/stromal cell recruitment. Sci. Rep. 7 (1), 1667. 10.1038/s41598-017-01809-x 28490808 PMC5431789

[B124] Solis A. G. Bielecki P. Steach H. R. Sharma L. Harman C. C. D. Yun S. (2019). Mechanosensation of cyclical force by PIEZO1 is essential for innate immunity. Nature 573 (7772), 69–74. 10.1038/s41586-019-1485-8 31435009 PMC6939392

[B125] Sonoda S. Yamaza H. Ma L. Tanaka Y. Tomoda E. Aijima R. (2016). Interferon-gamma improves impaired dentinogenic and immunosuppressive functions of irreversible pulpitis-derived human dental pulp stem cells. Sci. Rep. 6, 19286. 10.1038/srep19286 26775677 PMC4726054

[B126] Stirnemann J. Belmatoug N. Camou F. Serratrice C. Froissart R. Caillaud C. (2017). A review of gaucher disease pathophysiology, clinical presentation and treatments. Int. J. Mol. Sci. 18 (2), 441. 10.3390/ijms18020441 28218669 PMC5343975

[B127] Strojny C. Boyle M. Bartholomew A. Sundivakkam P. Alapati S. (2015). Interferon gamma-treated dental pulp stem cells promote human mesenchymal stem cell migration *in vitro* . J. Endod. 41 (8), 1259–1264. 10.1016/j.joen.2015.02.018 26051078 PMC4624205

[B128] Sugimoto A. Miyazaki A. Kawarabayashi K. Shono M. Akazawa Y. Hasegawa T. (2017). Piezo type mechanosensitive ion channel component 1 functions as a regulator of the cell fate determination of mesenchymal stem cells. Sci. Rep. 7 (1), 17696. 10.1038/s41598-017-18089-0 29255201 PMC5735093

[B129] Sun J. Wang Z. Liu P. Hu Y. Yang J. (2022). Exosomes derived from human gingival mesenchymal stem cells attenuate the inflammatory response in periodontal ligament stem cells. Front. Chem. 10, 863364. 10.3389/fchem.2022.863364 35464198 PMC9019468

[B130] Sun W. Chi S. Li Y. Ling S. Tan Y. Xu Y. (2019). The mechanosensitive Piezo1 channel is required for bone formation. eLife 8, e47454. 10.7554/eLife.47454 31290742 PMC6685704

[B131] Takayanagi H. (2007). Osteoimmunology: shared mechanisms and crosstalk between the immune and bone systems. Nat. Rev. Immunol. 7 (4), 292–304. 10.1038/nri2062 17380158

[B132] Tang M. Tian L. Luo G. Yu X. (2018). Interferon-Gamma-mediated osteoimmunology. Front. Immunol. 9, 1508. 10.3389/fimmu.2018.01508 30008722 PMC6033972

[B133] Tan J. Dai A. Pan L. Zhang L. Wang Z. Ke T. (2021). Inflamm-aging-related cytokines of IL-17 and IFN-γ accelerate osteoclastogenesis and periodontal destruction. J. Immunol. Res. 2021, 9919024. 10.1155/2021/9919024 34395635 PMC8357511

[B134] Tao D. Zhang L. Ding Y. Tang N. Xu X. Li G. (2023). Primary cilia support cartilage regeneration after injury. Int. J. Oral Sci. 15 (1), 22. 10.1038/s41368-023-00223-6 37268650 PMC10238430

[B135] Tatullo M. Marrelli M. Shakesheff K. M. White L. J. (2015). Dental pulp stem cells: function, isolation and applications in regenerative medicine. J. tissue Eng. Regen. Med. 9 (11), 1205–1216. 10.1002/term.1899 24850632

[B136] Thielen N. G. M. Van Der Kraan P. M. Van Caam A. P. M. (2019). TGFβ/BMP signaling pathway in cartilage homeostasis. Cells 8 (9), 969. 10.3390/cells8090969 31450621 PMC6769927

[B137] Tomokiyo A. Wada N. Maeda H. (2019). Periodontal ligament stem cells: regenerative potency in periodontium. Stem cells Dev. 28 (15), 974–985. 10.1089/scd.2019.0031 31215350

[B138] Tsukasaki M. Takayanagi H. (2019). Osteoimmunology: evolving concepts in bone-immune interactions in health and disease. Nat. Rev. Immunol. 19 (10), 626–642. 10.1038/s41577-019-0178-8 31186549

[B139] Tyagi A. M. Yu M. Darby T. M. Vaccaro C. Li J. Y. Owens J. A. (2018). The microbial metabolite butyrate stimulates bone formation via T regulatory cell-mediated regulation of WNT10B expression. Immunity 49 (6), 1116–1131. 10.1016/j.immuni.2018.10.013 30446387 PMC6345170

[B140] Valverde Lde F. Pereira T. D. E. A. Dias R. B. Guimarães V. S. N. Ramos E. A. G. Santos J. N. (2016). Macrophages and endothelial cells orchestrate tumor-associated angiogenesis in oral cancer via hedgehog pathway activation. Tumour Biol. 37 (7), 9233–9241. 10.1007/s13277-015-4763-6 26768620

[B141] Van Der Horst G. Farih-Sips H. LöWIK C. W. Karperien M. (2003). Hedgehog stimulates only osteoblastic differentiation of undifferentiated KS483 cells. Bone 33 (6), 899–910. 10.1016/j.bone.2003.07.004 14678849

[B142] Von Moltke J. Ayres J. S. Kofoed E. M. Chavarría-Smith J. Vance R. E. (2013). Recognition of bacteria by inflammasomes. Annu. Rev. Immunol. 31, 73–106. 10.1146/annurev-immunol-032712-095944 23215645

[B143] Wang D. Lyu Y. Yang Y. Zhang S. Chen G. Pan J. (2022). Schwann cell-derived EVs facilitate dental pulp regeneration through endogenous stem cell recruitment via SDF-1/CXCR4 axis. Acta biomater. 140, 610–624. 10.1016/j.actbio.2021.11.039 34852303

[B144] Wang F. Yu M. Yan X. Wen Y. Zeng Q. Yue W. (2011). Gingiva-derived mesenchymal stem cell-mediated therapeutic approach for bone tissue regeneration. Stem cells Dev. 20 (12), 2093–2102. 10.1089/scd.2010.0523 21361847

[B145] Wang H. Zhang H. Wang Y. Brown Z. J. Xia Y. Huang Z. (2021). Regulatory T-cell and neutrophil extracellular trap interaction contributes to carcinogenesis in non-alcoholic steatohepatitis. J. hepatology 75 (6), 1271–1283. 10.1016/j.jhep.2021.07.032 PMC12888775 34363921

[B146] Wang L. You X. Lotinun S. Zhang L. Wu N. Zou W. (2020a). Mechanical sensing protein PIEZO1 regulates bone homeostasis via osteoblast-osteoclast crosstalk. Nat. Commun. 11 (1), 282. 10.1038/s41467-019-14146-6 31941964 PMC6962448

[B147] Wang M. Li J. Ye Y. Song J. (2020b). SHED-derived conditioned exosomes enhance the osteogenic differentiation of PDLSCs via Wnt and BMP signaling *in vitro* . . Differ. Res. Biol. Divers. 111, 1–11. 10.1016/j.diff.2019.10.003 31630077

[B148] Wang T. Li W. Zhang Y. Xu X. Qiang L. Miao W. (2023). Bioprinted constructs that simulate nerve-bone crosstalk to improve microenvironment for bone repair. Bioact. Mater. 27, 377–393. 10.1016/j.bioactmat.2023.02.013 37122897 PMC10131128

[B149] Wang Y. Yan M. Wang Z. Wu J. Zheng Y. (2013). Dental pulp stem cells from traumatically exposed pulps exhibited an enhanced osteogenic potential and weakened odontogenic capacity. Archives oral Biol. 58 (11), 1709–1717. 10.1016/j.archoralbio.2013.09.001 24112738

[B150] Wang Z. Ding L. Zheng X. L. Wang H. X. (2014). DC-derived exosomes induce osteogenic differentiation of mesenchymal stem cells. Zhongguo shi yan xue ye xue za zhi 22 (3), 600–604. 10.7534/j.issn.1009-2137.2014.03.005 24989261

[B151] Watanabe Y. Fukuda T. Hayashi C. Nakao Y. Toyoda M. Kawakami K. (2022). Extracellular vesicles derived from GMSCs stimulated with TNF-α and IFN-α promote M2 macrophage polarization via enhanced CD73 and CD5L expression. Sci. Rep. 12 (1), 13344. 10.1038/s41598-022-17692-0 35922474 PMC9349189

[B152] Weitzmann M. N. (2017). Bone and the immune system. Toxicol. Pathol. 45 (7), 911–924. 10.1177/0192623317735316 29046115 PMC5749254

[B153] Wong R. Smith C. J. Allan S. M. Pinteaux E. (2023). Preconditioning with interleukin-1 alpha is required for the neuroprotective properties of mesenchymal stem cells after ischaemic stroke in mice. J. Cereb. blood flow metabolism 43 (12), 2040–2048. 10.1177/0271678X231197109 PMC10925871 37602422

[B154] Xie X. Xu C. Zhao L. Wu Y. Feng J. Q. Wang J. (2022). Axin2-expressing cells in the periodontal ligament are regulated by bone morphogenetic protein signalling and play a pivotal role in periodontium development. J. Clin. periodontology 49 (9), 945–956. 10.1111/jcpe.13666 35634660

[B155] Xie Z. Tang S. Ye G. Wang P. Li J. Liu W. (2018). Interleukin-6/interleukin-6 receptor complex promotes osteogenic differentiation of bone marrow-derived mesenchymal stem cells. Stem Cell Res. Ther. 9 (1), 13. 10.1186/s13287-017-0766-0 29357923 PMC5776773

[B156] Xiong Y. Mi B. B. Lin Z. Hu Y. Q. Yu L. Zha K. K. (2022). The role of the immune microenvironment in bone, cartilage, and soft tissue regeneration: from mechanism to therapeutic opportunity. Mil. Med. Res. 9 (1), 65. 10.1186/s40779-022-00426-8 36401295 PMC9675067

[B157] Xu H. Guan J. Jin Z. Yin C. Wu S. Sun W. (2022). Mechanical force modulates macrophage proliferation via Piezo1-AKT-Cyclin D1 axis. FASEB J. 36 (8), e22423. 10.1096/fj.202200314R 35775626 PMC12166287

[B158] Yang N. Liu Y. (2021). The role of the immune microenvironment in bone regeneration. Int. J. Med. Sci. 18 (16), 3697–3707. 10.7150/ijms.61080 34790042 PMC8579305

[B159] Yang R. Yu T. Liu D. Shi S. Zhou Y. (2018). Hydrogen sulfide promotes immunomodulation of gingiva-derived mesenchymal stem cells via the Fas/FasL coupling pathway. Stem Cell Res. Ther. 9 (1), 62. 10.1186/s13287-018-0804-6 29523215 PMC5845196

[B160] Yang X. Zhou F. Yuan P. Dou G. Liu X. Liu S. (2021). T cell-depleting nanoparticles ameliorate bone loss by reducing activated T cells and regulating the Treg/Th17 balance. Bioact. Mater. 6 (10), 3150–3163. 10.1016/j.bioactmat.2021.02.034 33778195 PMC7970013

[B161] Yang Y. Wang X. Miron R. J. Zhang X. (2019). The interactions of dendritic cells with osteoblasts on titanium surfaces: an *in vitro* investigation. Clin. oral Investig. 23 (11), 4133–4143. 10.1007/s00784-019-02852-w 30850859

[B162] Yang Z. Ma L. Du C. Wang J. Zhang C. Hu L. (2023). Dental pulp stem cells accelerate wound healing through CCL2-induced M2 macrophages polarization. iScience 26 (10), 108043. 10.1016/j.isci.2023.108043 37829207 PMC10565783

[B163] Yan W. Lin X. Ying Y. Li J. Fan Z. (2023). Specific RNA m6A modification sites in bone marrow mesenchymal stem cells from the jawbone marrow of type 2 diabetes patients with dental implant failure. Int. J. Oral Sci. 15 (1), 6. 10.1038/s41368-022-00202-3 36631441 PMC9834262

[B164] Yao Z. Getting S. J. Locke I. C. (2021). Regulation of TNF-induced osteoclast differentiation. Cells 11 (1), 132. 10.3390/cells11010132 35011694 PMC8750957

[B165] Yu V. W. Saez B. Cook C. Lotinun S. Pardo-Saganta A. Wang Y. H. (2015). Specific bone cells produce DLL4 to generate thymus-seeding progenitors from bone marrow. J. Exp. Med. 212 (5), 759–774. 10.1084/jem.20141843 25918341 PMC4419348

[B166] Yuan X. Pei X. Zhao Y. Tulu U. S. Liu B. Helms J. A. (2018). A wnt-responsive PDL population effectuates extraction socket healing. J. Dent. Res. 97 (7), 803–809. 10.1177/0022034518755719 29420105 PMC6728586

[B167] Yuan Y. Zhang Q. Wu B. Huang T. Gong P. Xiang L. (2023). Oncostatin M regulates macrophages polarization in osseointegration via yes-associated protein. Int. Immunopharmacol. 120, 110348. 10.1016/j.intimp.2023.110348 37220694

[B168] Yu F. Lian R. Liu L. Liu T. Bi C. Hong K. (2022). Biomimetic hydroxyapatite nanorods promote bone regeneration via accelerating osteogenesis of BMSCs through T cell-derived IL-22. ACS Nano 16 (1), 755–770. 10.1021/acsnano.1c08281 35005890

[B169] Zarubova J. Hasani-Sadrabadi M. M. Dashtimoghadam E. Zhang X. Ansari S. Li S. (2022). Engineered delivery of dental stem-cell-derived extracellular vesicles for periodontal tissue regeneration. Adv. Healthc. Mater. 11 (12), e2102593. 10.1002/adhm.202102593 35191610 PMC9233004

[B170] Zeng W. Liu G. Luan Q. Yang C. Yu X. (2021). B-cell deficiency exacerbates inflammation and bone loss in ligature-induced experimental periodontitis in mice. J. Inflamm. Res. 14, 5367–5380. 10.2147/JIR.S330875 34703274 PMC8526950

[B171] Zhang Q. Z. Su W. R. Shi S. H. Wilder-Smith P. Xiang A. P. Wong A. (2010). Human gingiva-derived mesenchymal stem cells elicit polarization of m2 macrophages and enhance cutaneous wound healing. Stem cells Dayt. Ohio 28 (10), 1856–1868. 10.1002/stem.503 PMC3114043 20734355

[B172] Zhang B. Han F. Wang Y. Sun Y. Zhang M. Yu X. (2022). Cells-micropatterning biomaterials for immune activation and bone regeneration. Adv. Sci. Weinheim, Baden-Wurttemberg, Ger. 9 (18), e2200670. 10.1002/advs.202200670 PMC9218778 35478383

[B173] Zhang D. Lin W. Jiang S. Deng P. Liu L. Wang Q. (2023a). Lepr-expressing PDLSCs contribute to periodontal homeostasis and respond to mechanical force by Piezo1. Adv. Sci. Weinheim, Baden-Wurttemberg, Ger. 10 (29), e2303291. 10.1002/advs.202303291 PMC10582421 37553778

[B174] Zhang D. Zhang S. Wang J. Li Q. Xue H. Sheng R. (2020a). LepR-expressing stem cells are essential for alveolar bone regeneration. J. Dent. Res. 99 (11), 1279–1286. 10.1177/0022034520932834 32585118

[B175] Zhang F. Si M. Wang H. Mekhemar M. K. Dörfer C. E. Fawzy El-Sayed K. M. (2017a). IL-1/TNF-α inflammatory and anti-inflammatory synchronization affects gingival stem/progenitor cells' regenerative attributes. Stem cells Int. 2017, 1349481. 10.1155/2017/1349481 29250118 PMC5700502

[B176] Zhang J. Shi H. Zhang N. Hu L. Jing W. Pan J. (2020b). Interleukin-4-loaded hydrogel scaffold regulates macrophages polarization to promote bone mesenchymal stem cells osteogenic differentiation via TGF-β1/Smad pathway for repair of bone defect. Cell Prolif. 53 (10), e12907. 10.1111/cpr.12907 32951298 PMC7574882

[B177] Zhang X. Ning T. Wang H. Xu S. Yu H. Luo X. (2019). Stathmin regulates the proliferation and odontoblastic/osteogenic differentiation of human dental pulp stem cells through Wnt/β-catenin signaling pathway. J. proteomics 202, 103364. 10.1016/j.jprot.2019.04.014 31009804

[B178] Zhang X. Xiong Q. Lin W. Wang Q. Zhang D. Xu R. (2023b). Schwann cells contribute to alveolar bone regeneration by promoting cell proliferation. J. bone mineral Res. 38 (1), 119–130. 10.1002/jbmr.4735 36331097

[B179] Zhang Y. BöSE T. Unger R. E. Jansen J. A. Kirkpatrick C. J. van den Beucken J. J. J. P. (2017b). Macrophage type modulates osteogenic differentiation of adipose tissue MSCs. Cell tissue Res. 369 (2), 273–286. 10.1007/s00441-017-2598-8 28361303 PMC5552848

[B180] Zhao Z. Zhao Q. Chen H. Chen F. Wang F. Tang H. (2023). Role of dendritic cells in MYD88-mediated immune recognition and osteoinduction initiated by the implantation of biomaterials. Int. J. Oral Sci. 15 (1), 31. 10.1038/s41368-023-00234-3 37532700 PMC10397189

[B181] Zhao Z. Zhao Q. Gu B. Yin C. Shen K. Tang H. (2020). Minimally invasive implantation and decreased inflammation reduce osteoinduction of biomaterial. Theranostics 10 (8), 3533–3545. 10.7150/thno.39507 32206106 PMC7069090

[B182] Zheng J. Kong Y. Hu X. Li Z. Li Y. Zhong Y. (2020). MicroRNA-enriched small extracellular vesicles possess odonto-immunomodulatory properties for modulating the immune response of macrophages and promoting odontogenesis. Stem Cell Res. Ther. 11 (1), 517. 10.1186/s13287-020-02039-1 33256846 PMC7708107

[B183] Zheng X. Wang S. Xiao L. Han P. Xie K. Ivanovski S. (2022). LiCl-induced immunomodulatory periodontal regeneration via the activation of the Wnt/β-catenin signaling pathway. J. periodontal Res. 57 (4), 835–848. 10.1111/jre.13022 35675063 PMC9541255

[B184] Zheng Y. Dong C. Yang J. Jin Y. Zheng W. Zhou Q. (2019). Exosomal microRNA-155-5p from PDLSCs regulated Th17/Treg balance by targeting sirtuin-1 in chronic periodontitis. J. Cell. physiology 234 (11), 20662–20674. 10.1002/jcp.28671 31016751

[B185] Zhou M. Graves D. T. (2022). Impact of the host response and osteoblast lineage cells on periodontal disease. Front. Immunol. 13, 998244. 10.3389/fimmu.2022.998244 36304447 PMC9592920

[B186] Zhu B. Liu W. Liu Y. Zhao X. Zhang H. Luo Z. (2023). Retraction Note: jawbone microenvironment promotes periodontium regeneration by regulating the function of periodontal ligament stem cells. Sci. Rep. 13 (1), 10034. 10.1038/s41598-023-36331-w 37339998 PMC10281943

